# Chaperone-Mediated Regulation of Choline Acetyltransferase Protein Stability and Activity by HSC/HSP70, HSP90, and p97/VCP

**DOI:** 10.3389/fnmol.2017.00415

**Published:** 2017-12-12

**Authors:** Trevor M. Morey, Warren Winick-Ng, Claudia Seah, R. Jane Rylett

**Affiliations:** ^1^Molecular Medicine Research Laboratories, Robarts Research Institute, University of Western Ontario, London, ON, Canada; ^2^Department of Physiology and Pharmacology, Schulich School of Medicine & Dentistry, University of Western Ontario, London, ON, Canada; ^3^Epigenetic Regulation and Chromatin Architecture Group, Berlin Institute for Medical Systems Biology, Max-Delbrück Centre for Molecular Medicine, Berlin, Germany

**Keywords:** choline acetyltransferase, BioID, heat shock proteins, ubiquitination, CHIP/Stub1, proximity-ligation assay, Cdc48/p97/VCP

## Abstract

Choline acetyltransferase (ChAT) synthesizes the neurotransmitter acetylcholine in cholinergic neurons, and mutations of this enzyme are linked to the neuromuscular disorder congenital myasthenic syndrome (CMS). One CMS-related mutation, V18M, reduces ChAT enzyme activity and cellular protein levels, and is located within a highly-conserved N-terminal proline-rich motif at residues _14_PKLP*V*PP_20_. We showed previously that disruption of this proline-rich motif by either proline-to-alanine mutation (P17A/P19A) or mutation of residue Val^18^ (V18M) enhances ubiquitination and degradation of these mutant ChAT proteins expressed in cholinergic SN56 cells by an unknown mechanism. In this study, using proximity-dependent biotin identification (BioID), co-immunoprecipitation and *in situ* proximity-ligation assay (PLA), we identified the heat shock proteins (HSPs) HSC/HSP70 and HSP90 as novel ChAT protein-interactors. These molecular chaperones are well-known for promoting the folding and stabilization of cellular proteins. Thus, we found that inhibition of HSPs by treatment of cells with either the HSC/HSP70 inhibitors 2-phenylethynesulfonamide (PES) or VER-155008, or the HSP90 inhibitor 17-AAG reduced cellular ChAT activity and solubility, and enhanced the ubiquitination and proteasome-dependent loss of ChAT protein. Importantly, the effects of HSP inhibition were greater for mutant ChAT proteins (P17A/P19A-ChAT and CMS-related V18M- and A513T-ChAT) compared to wild-type ChAT. HSPs can promote ubiquitination and degradation of terminally misfolded proteins through cooperative interaction with the E3 ubiquitin ligase CHIP/Stub1, and while we show that ChAT interacts with CHIP *in situ*, siRNA-mediated knock-down of CHIP had no effect on either wild-type or mutant ChAT protein levels. However, inhibition of the endoplasmic reticulum (ER)- and HSP-associated co-chaperone p97/VCP prevented degradation of ubiquitinated ChAT. Together, these results identify novel mechanisms for the functional regulation of wild-type and CMS-related mutant ChAT by pro-stabilizing HSPs and the pro-degradative co-chaperone p97/VCP that may have broader implications for ChAT function during cellular stress and disease.

## Introduction

Choline *O*-acetyltransferase (ChAT, EC2.3.1.6) catalyzes synthesis of the neurotransmitter acetylcholine (ACh) using the substrates acetyl-CoA and choline (Oda, 1999; Abreu-Villaca et al., 2011). ChAT is a phenotypic marker of both central and peripheral cholinergic neurons where ACh is essential for behavioral, cognitive, autonomic and neuromuscular functions (Abreu-Villaca et al., 2011; Picciotto et al., 2012). To date, multiple ChAT transcripts have been identified that are formed by alternative splicing and differential use of the non-coding exons R, N, M, S, and H (Benjanin et al., 1994; Misawa et al., 1997; Oda, 1999; Oda et al., 2004). All transcripts encode a common 69-kDa ChAT enzyme that is localized predominantly to the neuronal cytoplasm where it generates ACh (Rylett and Schmidt, 1993; Misawa et al., 1997; Dobransky et al., 2001; Ohno et al., 2001). Additionally, in humans and non-human primates only, the M-transcript also encodes for an 82-kDa ChAT protein due to the presence of a unique in-frame translation initiation codon located 5′ to the common translation initiation site for 69-kDa ChAT (Oda et al., 1992; Chireux et al., 1995; Misawa et al., 1997; Ohno et al., 2001). This primate-specific 82-kDa ChAT protein contains a nuclear localization signal (NLS) located within its extended N-terminus that directs it to the nucleus of cholinergic neurons in human brain and spinal cord (Resendes et al., 1999; Gill et al., 2003, 2007). Though our lab has shown that nuclear 82-kDa ChAT can synthesize ACh (Resendes et al., 1999; Gill et al., 2003) and alter gene expression (Albers et al., 2014; Winick-Ng et al., 2016), the function/s of this ChAT protein remain under investigation.

Alterations in the catalytic activity of 69-kDa ChAT are found in a number of neurological disorders, including Alzheimer's disease (AD) (Pakaski and Kalman, 2008), amyotrophic lateral sclerosis (Kato, 1989) and Huntington's disease (Lange et al., 1992). Importantly, mutations in ChAT protein have been causally-linked to the development of congenital myasthenic syndrome (CMS), a rare and inheritable neuromuscular disorder in which synaptic mechanisms are compromised, resulting in chronic hypotonia and acute apnea (Engel et al., 2012, 2015). While mutations in proteins localized both pre- and postsynaptically at neuromuscular junctions are linked to CMS (Engel et al., 2012, 2015), *in vitro* electrophysiological studies of CMS patients revealed that ACh synthesis can be impaired directly (Engel et al., 1977; Engel and Lambert, 1987; Mora et al., 1987). Subsequently, over 40 CMS-related missense mutations have been identified in human 69-kDa ChAT protein, some of which have been partially characterized biochemically (Ohno et al., 2001; Byring et al., 2002; Kraner et al., 2003; Maselli et al., 2003; Schmidt et al., 2003; Barisic et al., 2005; Mallory et al., 2009; Yeung et al., 2009; Shen et al., 2011; Arredondo et al., 2015).

Severe CMS symptoms are associated with mutations involving the ChAT active-site that impair substrate-binding, reduce mutant ChAT protein levels and result in reduced ACh synthesis (Ohno et al., 2001; Shen et al., 2011; Arredondo et al., 2015). Interestingly, a V18M mutation identified in four unrelated patients with severe CMS is located distal to the ChAT active-site, but also reduces both ChAT protein levels and acetyl-CoA affinity and catalytic efficiency (Shen et al., 2011; Arredondo et al., 2015). Residue Val^18^ does not participate directly in acetyl-CoA binding, but is located within a highly-conserved, surface-exposed N-terminal proline-rich motif with sequence _14_PKLP*V*PP_20_ that shares homology with the core PxxP binding-motif for SH3-binding domains (Kim et al., 2006; Kurochkina and Guha, 2013). We showed recently that ChAT protein stability is regulated by the ubiquitin-proteasome system and, importantly, that mutation of this proline-rich motif, including P17A/P19A and CMS-related V18M, as well as the CMS-related mutant A513T enhances the ubiquitination and proteasome-dependent degradation of ChAT protein (Morey et al., 2016). The mechanisms responsible for the basal regulation of ChAT protein stability and/or for the changes observed following mutation of ChAT, in particular disruption of the N-terminal proline-rich motif, have not been elucidated.

Disease-related mutations can often lead to protein misfolding, dysfunction and enhanced degradation. Heat shock proteins (HSP), including HSP40, heat shock cognate 71 kDa protein (HSC70) and HSP90, act together as molecular chaperones to regulate the folding of non-native nascent proteins and to prevent protein misfolding and aggregation (McClellan and Frydman, 2001; Richter et al., 2010). Protein-damaging stresses, such as oxidative stress, can promote protein misfolding, dysfunction, and cell death (Kalmar and Greensmith, 2009; Vabulas et al., 2010). In response, stress-induced expression of HSP70 attenuates protein misfolding, stimulates refolding of stress-damaged proteins and prevents aggregation of misfolded proteins (Mosser et al., 1997). In neurodegenerative diseases, such as AD and Huntington's disease, HSPs can have a cytoprotective role by chaperoning disease-associated aggregation-prone proteins (Lackie et al., 2017). If protein misfolding is irreversible, such as during chronic or intense proteotoxic stress or in the case of mutant proteins, terminally misfolded proteins can be targeted for degradation by the HSP-associated co-chaperone C-terminus of HSC70-interacting protein (CHIP) (Ballinger et al., 1999; Connell et al., 2001; Zhang et al., 2015). CHIP is a member of the U-box family of E3 ubiquitin ligases that has neuroprotective properties by regulating the degradation of aggregation-prone tau protein (Petrucelli et al., 2004), as well as amyloid precursor protein (Kumar et al., 2007) and the β-amyloid (Aβ) promoting enzyme β-secretase-1 (Singh and Pati, 2015).

The goal of this study was to elucidate mechanisms involved in regulation of ChAT protein stability. By comparing wild-type ChAT with mutant ChAT proteins that we found previously to undergo enhanced ubiquitination (i.e., P17A/P19A-, V18M-, and A513T-ChAT) (Morey et al., 2016), we identified HSC/HSP70 and HSP90 as novel ChAT protein-interactors and show that pharmacological inhibition of these HSPs results in loss of cellular ChAT activity and enhanced ubiquitination and degradation of ChAT. ChAT interacts with the E3 ligase CHIP, though siRNA knock-down of CHIP has no effect on ChAT protein levels. Lastly, pharmacological inhibition of the endoplasmic reticulum (ER)- and HSP-associated co-chaperone p97/VCP prevents degradation of ubiquitinated ChAT. Collectively, this study identifies and characterizes the role of multiple molecular chaperones in ChAT protein stability that could have broader implications for dysregulation of ChAT under conditions of cellular stress or in human disease.

## Materials and methods

### Protein expression plasmids

Plasmids were constructed by ligating full-length cDNAs encoding wild-type human 69-kDa ChAT (Dobransky et al., 2003) or mutant ChAT (P17A/P19A-ChAT, V18M-ChAT, or A513T-ChAT) (Morey et al., 2016) to pcDNA3.1+ vector (Invitrogen) for expression in mammalian cells. For proximity-dependent biotin identification (BioID) experiments, pcDNA3.1/MCS-BirA(R118G)-HA (BirA^*^; Addgene #36047) (Roux et al., 2012) served as a template for the ligation of PCR-amplified wild-type and P17A/P19A-ChAT cDNA. For the heterologous expression of CHIP, a plasmid was kindly provided by Dr. Yosef Shaul (Weizmann Institute of Science) with permission by Dr. Cam Patterson (UNC School of Medicine). CHIP cDNA was PCR amplified with the addition of a C-terminal FLAG-tag and ligated to pcDNA3.1+. All expression plasmids were verified by full-length DNA sequencing prior to use.

### Cell culture, experimental treatments, and cell lysis

Cholinergic SN56 neural cells (gift from Dr. J.K. Blusztajn, Boston University) (Blusztajn et al., 1992) or HEK293 cells (ATCC) were grown as monolayers in DMEM supplemented with 5–10% FBS (Invitrogen) and 1% Pen-Strep at 37°C with 5% CO_2_. Prior to experimental treatments, cells were transiently transfected by Lipofectamine 3,000 (Invitrogen) at ~50% confluence for 18–24 h at 37°C with plasmids above. All laboratory procedures were conducted with Biosafety Approval BIO-RRI-0022.

The impact of inhibition of HSC/HSP70, HSP90 or p97/VCP function on ChAT steady-state protein levels was determined in SN56 cells expressing either wild-type or mutant ChAT proteins treated with varying concentrations of the HSC/HSP70 inhibitor VER-155008 (5–50 μM; Sigma), HSP90 inhibitor 17-AAG (0.5–2 μM; StressMarq Biosciences) or p97/VCP inhibitor Eeyarestatin-I (5–10 μM; Sigma) for 18–24 h at 37°C. Cells were collected and lysed on ice in RIPA buffer (50 mM Tris-HCl; pH 8.0, 150 mM NaCl, 1% Triton X-100, 0.5% sodium deoxycholate, 0.1% SDS) supplemented with mammalian protease inhibitor cocktail (Sigma), phosphatase inhibitor cocktail (10 mM NaF, 1 mM Na_3_VO_4_, 20 mM Na_2_HPO_4_, 3 mM β-glycerolphosphate, 5 mM sodium pyrophosphate), 50 μM MG132 (Enzo Life Sciences), 10 mM N-ethylmaleimide (NEM; Calbiochem) and 800 U/ml DNase I (Invitrogen). Lysates were centrifuged for 10 min at 21,000 g at 4°C, denatured in 1x reducing Laemmli sample buffer (63 mM Tris-HCl; pH 6.8, 10% glycerol, 2% SDS, 0.005% bromophenol blue, 2.5% 2-mercaptoethanol) at 95°C for 10 min, then analyzed by immunoblotting with anti-ChAT primary antibody.

The effects of either proteasome or lysosome inhibition on ChAT steady-state protein levels during HSC/HSP70 and HSP90 inhibition were investigated in SN56 cells expressing wild-type or mutant ChAT. Cells were treated either alone with 40 μM VER-155008 or 1 μM 17-AAG at 37°C for 24 h or co-treated for 18 h with either 5 μM MG132 or 50 μM chloroquine (Sigma). To lyse cells and release aggregated proteins following HSP inhibition, cells were boiled at 95°C for 5 min and sonicated in 1% SDS lysis buffer (50 mM Tris-HCl; pH 8.0, 150 mM NaCl, 1% SDS) supplemented with protease/phosphatase inhibitors, 50 μM MG132 and 10 mM NEM. Protein samples were prepared for anti-ChAT immunoblotting as above. Proteasome inhibition by MG132 treatment or lysosome inhibition by chloroquine treatment was validated by immunoblotting for the accumulation of either ubiquitinated cellular proteins or for a lysosome-associated isoform of microtubule-associated protein 1A/1B-light chain 3 (LC3B-II), respectively (Tanida et al., 2008).

### Cellular ChAT assay

To measure cellular ChAT activity following HSC/HSP70 or HSP90 inhibition SN56 cells transiently expressing wild-type ChAT were treated with 40 μM VER-155008 or 1 μM 17-AAG at 37°C for 24 h. Cells were collected and lysed on ice in buffer containing 10 mM Na_2_HPO_4_; pH 7.4, 0.87 mM EDTA, 0.5% Triton X-100, and 1.5 mM eserine sulfate, supplemented with protease/phosphatase inhibitors (above) and 50 μM MG132. Lysates were centrifuged for 10 min at 21,000 g at 4°C and cellular ChAT activity was analyzed by modified radioenzymatic assay (Fonnum, 1969; Pongrac and Rylett, 1998).

### Immunoprecipitation (IP) to assess ubiquitination of ChAT

SN56 cells transfected to express either wild-type or mutant ChAT were treated at 37°C with 40 μM VER-155008 for 24 h, 1 μM 17-AAG for either 8 or 24 h, or 10 μM Eeyarestatin-I for 18 h to inhibit HSC/HSP70, HSP90 or p97/VCP respectively. To block degradation of ubiquitinated ChAT during HSP inhibition cells were co-treated with 20 μM MG132 for 6 h prior to being collected; MG132 was omitted during assessment of ChAT ubiquitination following p97/VCP inhibition. Cells were lysed on ice in RIPA buffer supplemented with 0.5% SDS, protease/phosphatase inhibitors (above), 50 μM MG132 and 10 mM NEM. Lysates were sonicated, centrifuged for 15 min at 21,000 g at 4°C, then aliquots of cleared whole cell lysates were incubated with 25 μg/mg cellular protein of anti-ChAT primary antibody (CTab) (Dobransky et al., 2003) at 4°C for 18 h. Immune complexes were captured onto protein-G Dynabeads (Invitrogen) for 1 h at 4°C, washed with RIPA buffer with 0.5% SDS, then eluted into 50 μl of 2x Laemmli sample buffer at 85°C for 15 min. IP samples were analyzed by immunoblotting with anti-ChAT and anti-ubiquitin primary antibodies. Total protein samples were prepared for immunoblotting by denaturing whole cell lysates in 1x Laemmli sample buffer at 95°C for 10 min.

### Co-immunoprecipitation (co-IP) of ChAT with CHIP or HSPs

For co-IP of ChAT with endogenous HSPs, endogenous CHIP or heterologous FLAG-tagged CHIP either HEK293 or SN56 cells were transfected to express either wild-type or mutant ChAT protein alone or co-expressed with FLAG-CHIP. Cells were collected on ice and lysed in co-IP lysis buffer (10 mM HEPES; pH 7.4, 5 mM MgCl_2_, 1 mM EGTA, 50 mM NaCl, 5% glycerol, 0.5% Triton X-100) supplemented with protease/phosphatase inhibitors, 50 μM MG132 and 10 mM NEM. Lysates were centrifuged for 15 min at 15,000 g at 4°C, then aliquots of cleared whole cell lysates containing either 2 mg (FLAG-CHIP) or 4 mg (endogenous proteins) of protein were incubated with CTab primary antibody at 4°C for 18 h as above. Immune complexes were captured onto protein-G Dynabeads for 1 h at 4°C, washed in co-IP lysis buffer, then eluted into 50 μl of 2x Laemmli sample buffer at 85°C for 15 min. Co-IP samples were analyzed by immunoblotting. Total protein samples were prepared by denaturing whole cell lysates in 1x Laemmli sample buffer at 95°C for 10 min prior to immunoblotting.

### SDS-PAGE and immunoblotting analysis

Denatured protein samples from whole cell lysates and anti-ChAT IP/co-IPs were resolved on 7.5–15% SDS-PAGE gels, then transferred to PVDF membranes (Bio-Rad). For immunoblotting, membranes were blocked for 1 h at room temperature in 5% non-fat milk powder in PBS containing 0.15% Triton X-100 (PBST), followed by incubation overnight at 4°C with primary antibody. Probed membranes were washed with PBST, and primary antibodies were detected using 1:10,000 peroxidase-coupled secondary antibodies (Jackson ImmunoResearch) and Clarity Western ECL Substrate (Bio-Rad) on a ChemiDoc MP system (Bio-Rad). The following primary antibodies were used: 1:1,000 ChAT (CTab) (Dobransky et al., 2003), 1:10,000 β-actin (Sigma), 1:10,000 GAPDH (Cell Signalling), 1:1,000 HSC70 (StressMarq), 1:2,000 HSP70 (Thermo), 1:2,000 HSP90 (StressMarq), 1:1,000 CHIP (Santa Cruz), 1:1,000 ubiquitin (Santa Cruz), 1:1,000 FLAG-M2 (Sigma), and 1:1,000 LC3B-I/II (Thermo). For anti-CHIP immunoblotting from anti-ChAT co-IP samples, 1:1,000 VeriBlot IP secondary antibody (Abcam) was used to reduce IgG light chain interference from detecting endogenous 35-kDa CHIP.

### Triton X-100 fractionation of cells for soluble/insoluble ChAT and SDD-AGE

ChAT protein solubility was assessed in ChAT-expressing SN56 cells following treatment with 20 μM 2-phenylethynesulfonamide (PES; Sigma) at 37°C for 24 h. Cells were collected and lysed on ice in 0.1% Triton X-100 lysis buffer (50 mM Tris-HCL; pH 8.0, 150 mM NaCl, 0.1% Triton X-100) supplemented with protease/phosphatase inhibitors, 50 μM MG132 and 10 mM NEM. Lysates were centrifuged for 15 min at 15,000 g at 4°C and aliquots of Triton-soluble supernatant were prepared for immunoblotting by denaturing in 1x Laemmli sample buffer at 95°C for 10 min. Triton-insoluble proteins were prepared for immunoblotting by washing Triton-insoluble pellets once with ice-cold PBS, then denaturing the pellets in an equal volume of 2x Laemmli sample buffer with 5% 2-mercaptoethanol at 85°C for 15 min prior to separation on SDS-PAGE gels. Additionally, Triton-insoluble proteins were analyzed in parallel by semi-denaturing detergent agarose gel electrophoresis (SDD-AGE) to determine if PES treatment results in generation of SDS-resistant ChAT aggregates. Briefly, Triton-insoluble samples resuspended in 2x Laemmli sample buffer with 5% 2-mercaptoethanol were resolved on 1.8% agarose gels containing 0.5% SDS in 1x TAE buffer (40 mM Tris; pH 7.6, 20 mM acetic acid, 1 mM EDTA) supplemented with 0.1% SDS. Resolved proteins were transferred to PVDF membranes by overnight capillary transfer in 1x TBS with 10% methanol, then immunoblotted for ChAT protein as above.

### Proximity-dependent biotin identification (BioID)

HEK293 cells expressing wild-type- or P17A/P19A-ChAT-BirA^*^ fusion proteins were live-labeled with 50 μM biotin at 37°C for 24 h to allow proximity-dependent biotinylation of cellular proteins. Controls cells were transfected with either empty vector or plasmids encoding untagged ChAT or BirA^*^ (Roux et al., 2012). Cells were collected on ice and lysed in supplemented RIPA buffer (above), centrifuged for 10 min at 21,000 g at 4°C, then aliquots of cleared whole cell lysate containing 5 mg protein were incubated with 50 μl of MyOne Streptavidin C1 Dynabeads (Invitrogen) at 4°C for 18 h. Streptavidin pull-down (PD) samples were washed with RIPA buffer and biotinylated proteins were eluted into 50 μl of 2x Laemmli sample buffer at 85°C for 15 min prior to immunoblotting with 1:20,000 Pierce High Sensitivity Streptavidin-HRP (Thermo) in PBST with 5% BSA. Total protein samples were prepared by denaturing whole cell lysates in 1x Laemmli sample buffer at 95°C for 10 min prior to immunoblotting. For large-scale BioID to identify novel ChAT protein-interactors, HEK293 cells expressing either untagged ChAT (control) or ChAT-BirA^*^ fusion proteins were live labeled with 50 μM biotin then collected, lysed and centrifuged as above. Aliquots of cleared whole cell lysate containing 25 mg protein were incubated with 250 μl of MyOne Streptavidin C1 Dynabeads at 4°C for 18 h. Streptavidin PD samples were washed, eluted into 100 μl of 2x Laemmli sample buffer, and run on 10% SDS-PAGE gels. To visualize biotinylated proteins, the SDS-PAGE gel was stained using Pierce Silver Stain for Mass Spectrometry (Thermo).

### Mass spectrometry

Following large-scale BioID proteins of interest were excised from silver-stained SDS-PAGE gels using an Ettan Spot Picker (GE Healthcare Life Sciences). In-gel digestion was performed at the London Regional Proteomics Center - Functional Proteomics Facility (FPF; Western University) using a MassPREP automated digester station (PerkinElmer) where gel pieces were first de-stained [50 mM Na_2_S_2_O_3_ and 15 mM K_3_Fe(CN)_6_], followed by protein reduction (10 mM dithiothreitol), alkylation (55 mM iodoacetamide), and tryptic digestion. Digested peptides were extracted in 1% formic acid with 2% acetonitrile, lyophilized, then dissolved in a 10% acetonitrile containing 0.1% trifluoroacetic acid prior to mass spectrometry by either matrix-assisted laser desorption/ionization time-of-flight mass spectrometry (MALDI-TOF-MS) or by liquid chromatography-electrospray ionization-tandem mass spectrometry (LC-ESI-MS/MS).

For MALDI-TOF-MS, tryptic-digested peptides were mixed 1:1 (v/v) with a 5 mg/mL α-cyano-4–hydroxycinnamic acid MALDI matrix that was prepared in 6 mM NH_4_H_2_PO_4_, 50% acetonitrile, and 0.1% trifluoroacetic acid, then submitted to the London Regional Proteomics Center - FPF (Western University) for analysis. Mass spectrum were obtained using a TOF/TOF 5800 System (Applied Biosystems, Sciex) equipped with a 349 nm OptiBeam On-Axis laser using a laser pulse-rate of 400 Hz. Reflectron positive mode was used and was externally calibrated at 50 ppm mass tolerance and internally at 10 ppm. Each mass spectrum was collected as a sum of 500 shots. Data acquisition and processing were completed using a TOF/TOF Series Explorer and Data Explorer respectively (Applied Biosystems, Sciex). The resulting data were analyzed using the online Mascot Server (Matrix Science) against the NCBI Human protein database with the following selected as variable modifications; biotin (K), carbamidomethyl (C), NEM (C), oxidation (M). Peptide tolerance was set at ± 50 ppm for protein identification.

For LC-ESI-MS/MS, tryptic-digested peptides were submitted for protein identification by the London Regional Proteomics Center–Biological Mass Spectrometry Laboratory (BMSL; Western University). Briefly, digested peptides were injected into and analyzed using a LC-ESI-MS/MS system comprised of a nanoAcquity UPLC (Waters) followed by an in-line QExactive Hybrid Quadrupole-Orbitrap mass spectrometer (Thermo) using a top-12 double-play method. For LC, peptides were trapped onto a reverse-phase Acquity Symmetry C18 column, 5 μm, 180 μm × 200 mm (Waters), and eluted/separated from a Acquity Peptide BEH C18 column, 1.7 um, 75 um × 250 mm (Waters), and using and a H_2_O-acetonitrile with 0.1% formic acid solvent gradient. Resulting data were analyzed using Peaks 7.5 software (Bioinformatics Solutions Inc.,) against the NCBI Human protein database allowing for variable carbamidomethylation or modification of cysteine residues with NEM with filter criteria of FDR = 1% and UP = 1 (unique protein) to show protein identification.

### *In situ* proximity ligation assay (PLA)

To evaluate *in situ* ChAT protein-interactors, SN56 cells were plated on 35 mm glass-bottom confocal dishes and transfected to express wild-type ChAT. Cells were washed with HBSS, formalin-fixed (4% paraformaldehyde in HBSS) for 15 min, permeabilized with 0.1% Triton X-100, blocked for 1 h in HBSS supplemented with 3% donkey serum, then finally incubated for 1 h with primary antibodies targeting ChAT (1:100; Chemicon, goat primary) together with either endogenous HSC70 (1:100; StressMarq, mouse primary), HSP90 (1:200; StressMarq, mouse primary) or CHIP (1:200; Santa Cruz, rabbit primary); all steps were performed at room temperature. Controls cells were transfected with empty vector or were not incubated with primary antibodies. To visualize *in situ* interactions, we utilized DuoLink PLA technology (Sigma) by incubating cells with Duolink oligonucleotide-linked secondary antibodies (anti-goat PLUS with anti-mouse MINUS or anti-rabbit MINUS) for 1 h at 37°C. Cells were then washed and incubated with 1 U/μL DNA ligase followed by 10 U/μL DNA polymerase according to manufacturer's instructions. Finally, cells were overlaid with Duolink *In Situ* Mounting Medium with DAPI and images were acquired using a Leica True Confocal Scanner (TCS) 8 SpectroPhotometer (SP8; Leica Microsystems Inc.,) confocal laser microscope with a 63x-magnification oil-immersion objective (NA = 1.4), and processed using ImageJ 1.50 b software (NIH).

### Small-interfering RNA (siRNA) knock-down of CHIP

SN56 cells were incubated for a total of 72 h at 37°C with 25 nM of either scramble-control siRNA (Silencer Select #4390843; Thermo) or anti-Stub1/CHIP siRNA (Silencer Select ID: s80537; Thermo) following transfection with siLentFect Lipid Reagent (Bio-Rad). As a control, cells were mock transfected with siLentFect alone. After 24 h of CHIP knock-down, cells were transfected with Lipofectamine 3,000 to transiently express either wild-type or mutant ChAT for an additional 48 h at 37°C. Cells were collected and lysed on ice in RIPA buffer containing 0.5% SDS, protease/phosphatase inhibitors, 50 μM MG132 and 10 mM NEM. Lysates were centrifuged for 10 min at 21,000 g at 4°C, denatured in 1x Laemmli sample buffer at 95°C for 10 min, then analyzed by immunoblotting.

### Data analysis

For immunoblots from cell lysates, immunoreactive protein levels were measured by densitometry using ImageLab 5.0 software (Bio-Rad), then normalized to either β-actin or GAPDH protein levels in the same sample and graphed as mean ± SEM from *n* independent replicate experiments. For analysis of anti-ChAT co-IP immunoblots, individual immunoreactive bands for each co-immunoprecipitated protein (i.e. wild-type and mutant ChAT proteins, HSC/HSP70, HSP90 or FLAG-CHIP) were measured by densitometry then normalized to the total densitometric value for the protein on that immunoblot to obtain the relative levels of each co-immunoprecipitated protein. These relative levels of HSPs or FLAG-CHIP were then expressed as a function of the relative level of co-immunoprecipitated ChAT; data were graphed as mean ± SEM from *n* independent replicate co-IP experiments. Statistical analysis was performed by either Student's *t*-test, one-way ANOVA with either Tukey's or Dunnett's *post-hoc* test, or by two-way ANOVA with Bonferroni's *post-hoc* test using GraphPad Prism software.

## Results

### Identification of the heat shock proteins HSP70 and HSP90 as putative ChAT protein-interactors

**Figure 1:**
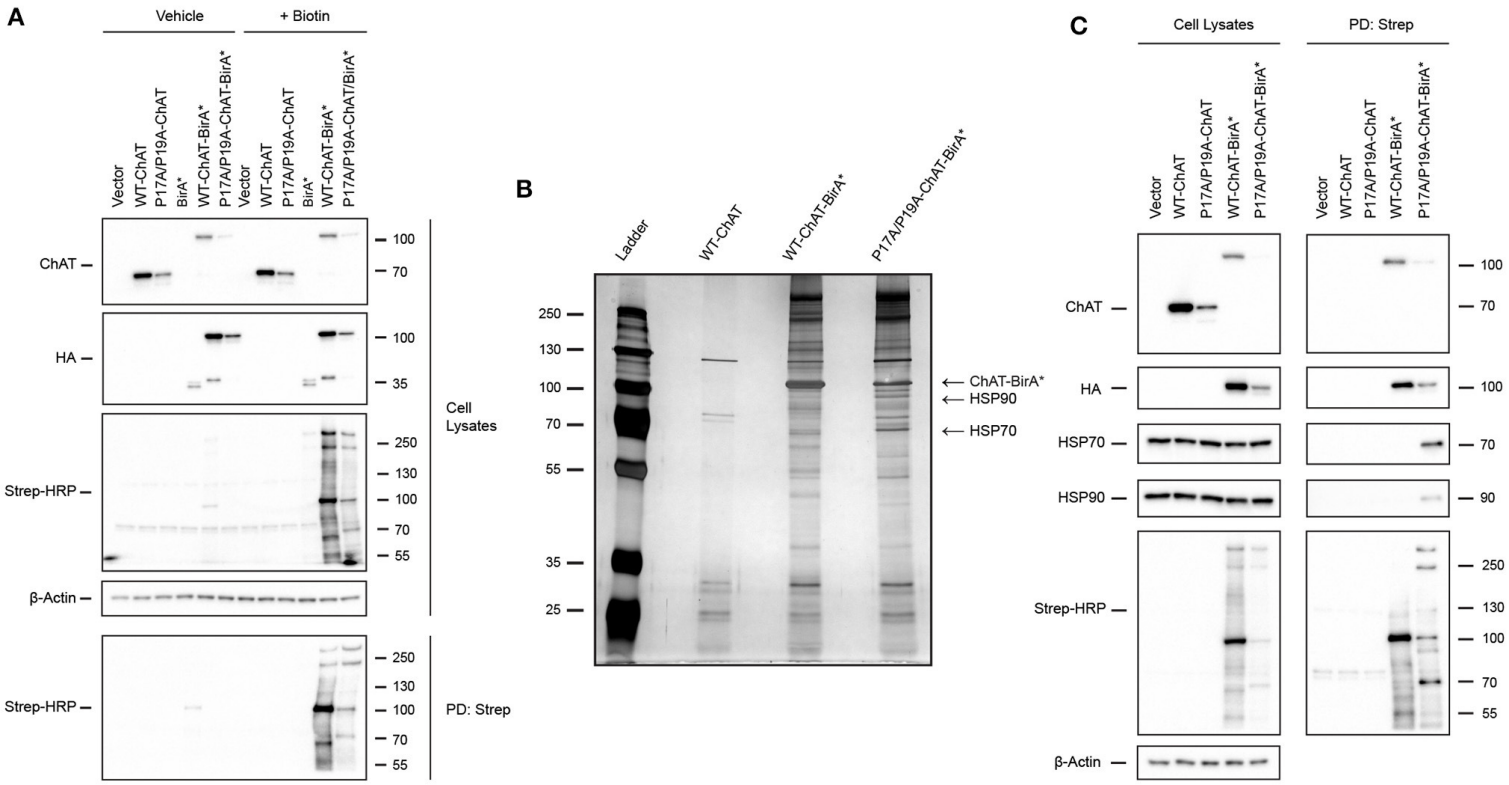
Identification of the heat shock proteins HSP70 and HSP90 as putative ChAT protein-interactors by proximity-dependent biotin identification (BioID). **(A)** Optimization of BioID in HEK293 cells expressing wild-type or P17A/P19A-ChAT fused to the HA-tagged promiscuous biotin ligase BirA-R118G (BirA^*^). Control cells were transfected with empty vector or plasmids encoding either untagged ChAT or BirA^*^. Cells were treated for 24 h with either 50 μM biotin to facilitate proximity-dependent biotinylation of ChAT-interacting cellular proteins or with vehicle-control (water). Biotinylated proteins were isolated from cell lysates by streptavidin pull-downs (PD: Strep) and immunoblotted as indicated (*n* = 2). **(B)** Identification of HSP70 and HSP90 as ChAT proximally-interacting proteins. Streptavidin PD samples prepared from biotin-treated HEK293 cells expressing wild-type-ChAT-BirA^*^ or P17A/P19A-ChAT-BirA^*^ fusion proteins were resolved and visualized on a silver-stained SDS-PAGE gels. Two proteins (~70 and ~90 kDa) that were enriched in samples expressing P17A/P19A-ChAT-BirA^*^ were identified by MALDI-TOF-MS or LC-ESI-MS/MS as HSP70 and HSP90, respectively. Control cells were transfected to express untagged wild-type ChAT (*n* = 1). **(C)** Confirmation of endogenous HSP70 and HSP90 as putative ChAT-interacting proteins by immunoblotting of streptavidin PD samples prepared from biotin-treated HEK293 cells expressing HA-tagged wild-type-ChAT-BirA^*^ or P17A/P19A-ChAT-BirA^*^. Control cells were transfected with empty vector or vector encoding untagged ChAT (*n* = 4).

We showed previously that mutations of the N-terminal proline-rich motif of ChAT, including P17A/P19A and CMS-related V18M, enhances ChAT ubiquitination and proteasomal degradation (Morey et al., 2016). To address the underlying mechanisms of ChAT ubiquitination and to identify putative protein-interactors that may differentially regulate mutant ChAT protein stability, we utilized the BioID proteomics approach (Roux et al., 2012) to screen for direct and proximal ChAT-interacting proteins (Figure 1). We chose to compare wild-type ChAT and P17A/P19A-ChAT for our BioID experiments as we showed previously that this mutation has the greatest impact on ChAT ubiquitination and degradation following modification of the proline-rich motif (Morey et al., 2016). Thus, HEK293 cells expressing either wild-type or P17A/P19A-ChAT fused to the 35 kDa HA-tagged promiscuous biotin ligase BirA-R118G-HA (BirA^*^) were treated with either vehicle (control) or 50 μM biotin for 24 h to allow proximity-dependent biotinylation of ChAT-interacting proteins. Anti-ChAT and anti-HA immunoblots revealed expression of ChAT-BirA^*^ fusion proteins with apparent molecular mass of ~100 kDa compared to untagged ChAT proteins at ~70 kDa (Figure 1A, top two panels). The steady-state levels of P17A/P19A-ChAT-BirA^*^ protein was less than that of wild-type-ChAT-BirA^*^ in agreement with our previous results demonstrating enhanced ubiquitination and degradation of mutant P17A/P19A-ChAT (Morey et al., 2016). Furthermore, the BioID-dependent biotinylation of cellular proteins was analyzed by blotting of samples obtained from streptavidin pull-downs (PDs) with HRP-conjugated streptavidin (Strep-HRP). As anticipated, we detected biotinylation of multiple cellular proteins in samples from biotin-treated HEK293 cells that expressed ChAT-BirA^*^ fusion proteins, but not in control cells grown in the absence of biotin or in cells expressing untagged ChAT (Figure 1A). Importantly, the abundance of several of these biotinylated proteins was different in cells expressing P17A/P19A-ChAT-BirA^*^ compared to wild-type-ChAT-BirA^*^; a protein with apparent molecular mass of ~70 kDa protein was notably enriched in cells expressing P17A/P19A-ChAT-BirA^*^.

Our initial BioID experiments suggest that multiple proteins interact with and may differentially regulate mutant P17A/P19A-ChAT compared to wild-type ChAT. Our primary goal for using BioID was to identify putative ChAT protein-interactors that may be responsible for the enhanced ubiquitination and degradation of mutant ChAT that we observed previously, and thus identification of proteins enriched in cells expressing P17A/P19A-ChAT-BirA^*^ were of prime interest. Therefore, we completed a large-scale BioID experiment in HEK293 cells expressing either wild-type-ChAT-BirA^*^ or P17A/P19A-ChAT-BirA^*^ and, following streptavidin PDs, biotinylated proteins were visualized on a silver stained SDS-PAGE gel (Figure 1B). Two proteins of interest that were enriched in cells expressing P17A/P19A-ChAT-BirA^*^ with apparent molecular masses of ~70 kDa and ~90 kDa were excised from the gel, along with an intense band at ~100 kDa correlating with the estimated molecular mass of the putative P17A/P19A-ChAT-BirA^*^ fusion protein. In-gel protein samples were digested with trypsin and identified by either matrix-assisted laser desorption/ionization time-of-flight mass spectrometry (MALDI-TOF-MS) and/or liquid chromatography-electrospray ionization-tandem mass spectrometry (LC-ESI-MS/MS). Initial identification by MALDI-TOF-MS revealed the ~70 kDa protein as the heat shock protein HSP70 and, as anticipated, the ~100 kDa protein as ChAT-BirA^*^ (Figure 1B). Sequence coverage obtained by MALDI-TOF-MS for human HSP70 was 30% (20 peptides; Supplementary Figure S1), while coverage for human 69-kDa ChAT and bacterial BirA were 31% (20 peptides) and 42% (16 peptides), respectively (Supplementary Figure S2). The ~100 kDa protein analyzed from cells expressing P17A/P19A-ChAT-BirA^*^ was identified from the NCBI protein database as ChAT-BirA^*^. Our data did not contain peptides with masses that matched tryptic peptides that would cover the mutated residues in the P17A/P19A-ChAT sequence. Identification of the ~90 kDa protein could not be achieved by MALDI-TOF-MS due to low sample yield, though subsequently by using the more sensitive LC-ESI-MS/MS approach we identified this protein as HSP90 with a 16% sequence coverage (10 peptides; Supplementary Figure S3). Also using LC-ESI-MS/MS, we obtained a greater sequence coverage for HSP70 of 34% (25 peptides; Supplementary Figure S4). Notably, tryptic peptides with masses indicating the addition of biotin were detected for both HSP70 and ChAT-BirA^*^ by MALDI-TOF-MS; these peptides contained lysine residues that could be biotinylated by BirA^*^.

To verify the proteins identified by MALDI and LC-ESI-MS/MS following BioID, we performed anti-HSP70 and anti-HSP90 immunoblotting on biotinylated protein samples obtained by streptavidin PDs from biotin-treated HEK293 cells expressing either wild-type-ChAT-BirA^*^ or P17A/P19A-ChAT-BirA^*^ (Figure 1C). Importantly, as anticipated we observed PD of endogenous HSP70 and HSP90 from biotin-treated cells expressing P17A/P19A-ChAT-BirA^*^; streptavidin PD of these HSPs were not consistently observed from cells expressing wild-type-ChAT-BirA^*^ (2 of 4 trials). As controls, neither HSP70 nor HSP90 were visualized in immunoblots following streptavidin PDs from cells that expressed untagged ChAT. In addition, anti-ChAT and anti-HA immunoblots revealed recovery of ChAT-BirA^*^, but not untagged ChAT, in streptavidin PD samples, suggesting that ChAT-BirA^*^ can undergo self-biotinylation. This has been reported in other BioID studies for BirA^*^-tagged proteins (Roux et al., 2012) and confirms our MALDI-TOF-MS analysis of biotinylated P17A/P19A-ChAT-BirA^*^ (Supplementary Figure S2). Together, our BioID results have identified the molecular chaperones HSP70 and HSP90 as putative ChAT protein-interactors that are enriched in cells expressing P17A/P19A-ChAT-BirA^*^.

### Co-IP of ChAT with HSPs is altered by mutation of N-terminal proline-rich motif of ChAT

**Figure 2:**
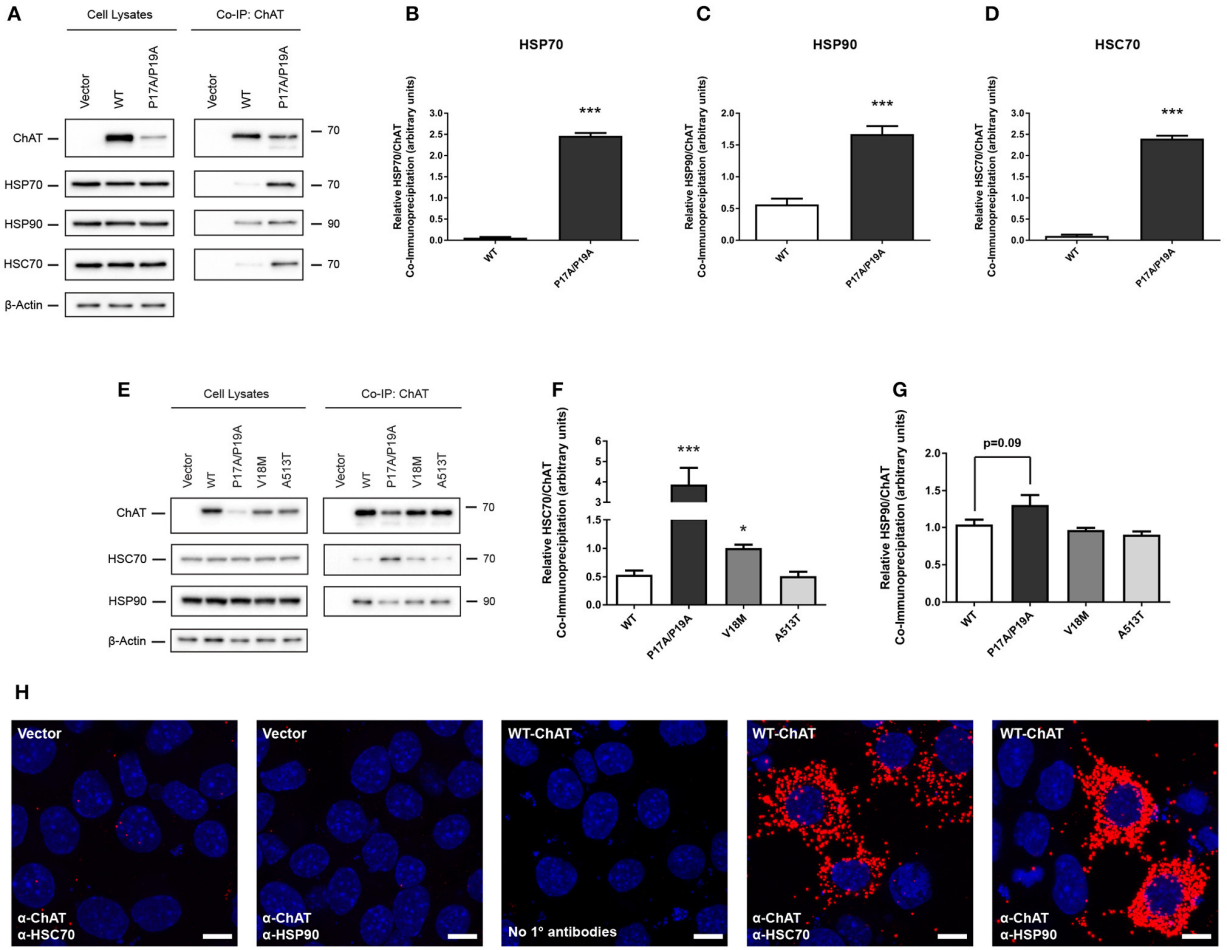
Co-immunoprecipitation (co-IP) of ChAT with heat shock proteins HSC70, HSP70, and HSP90 is altered by mutation of N-terminal proline-rich motif in ChAT. **(A)** Immunoblots showing co-IP of ChAT with endogenous HSC70, HSP70 and HSP90 from HEK293 cells expressing either wild-type or P17A/P19A-ChAT. Control cells were transfected with empty vector. Using HEK293 cells, co-IP of P17A/P19A-ChAT with HSP70 **(B)**, HSP90 **(C)** and HSC70 **(D)**, respectively, is greater than that of wild-type ChAT (^***^p ≤ 0.001, Student's *t*-test, mean ± SEM, *n* = 4). **(E)** Co-IP of ChAT with endogenous HSC70 and HSP90 from mouse cholinergic SN56 cells expressing either wild-type or P17A/P19A-ChAT or CMS-related mutant proteins V18M- or A513T-ChAT. Control cells were transfected with empty vector. **(F)** Using SN56 cells, Co-IP of P17A/P19A-ChAT (^***^p ≤ 0.001) and V18M-ChAT (^*^p ≤ 0.05), but not A531T-ChAT, with HSC70 is greater than that of wild-type ChAT (mean ± SEM, *n* = 5). **(G)** While there was a trend toward increased HSP90 interaction with P17A/P19A-ChAT (*p* = 0.09), no significant differences were observed for HSP90 interaction with mutant ChAT compared to wild-type ChAT in SN56 cells (mean ± SEM, *n* = 5). Statistical analysis for **(F)** and **(G)** was performed by one-way ANOVA with Dunnett's *post-hoc* test. **(H)** Detection of *in situ* interactions of wild-type ChAT with endogenous HSC70 and HSP90 by proximity ligation assay (PLA) in SN56 cells. Formalin-fixed cells were first co-labeled with goat anti-ChAT together with either mouse anti-HSC70 or mouse anti-HSP90 primary antibodies, then incubated with oligonucleotide-linked secondary antibodies. Following DNA ligation and DNA amplification using the Duolink *in Situ* Orange Kit (Sigma), *in situ* ChAT-HSP interactions were imaged by confocal microscopy. Positive *in situ* ChAT-HSP interactions where visualized as fluorescent red dots while nuclei were stained with DAPI (blue). Control cells were either transfected with empty vector or had primary antibodies omitted from the assay (No 1° antibodies). Images are representative of 3 independent experiments; scale bars are 10 μm.

HSPs, such as HSP70 and HSP90, are well-characterized molecular chaperones capable of performing protein triage decisions by either promoting the folding and stabilization of cellular proteins or by targeting terminally misfolded proteins for degradation (McClellan and Frydman, 2001; Richter et al., 2010). As such, these HSPs may represent molecular chaperones involved in the stabilization of ChAT and/or may promote degradation of mutant or misfolded ChAT. Therefore, to verify these putative BioID interactions between ChAT and endogenous HSP70 and HSP90, we performed co-IP assays from HEK293 cells expressing either untagged wild-type or P17A/P19A-ChAT (Figure 2A). Interestingly, both HSP70 and HSP90 were recovered in anti-ChAT co-IP samples from cells expressing either wild-type or P17A/P19A-ChAT. We also found that endogenous HSC70, a constitutively-expressed HSP that has analogous chaperone function and shares similar client proteins to that of HSP70 (Liu et al., 2012) co-IPs with ChAT (Figure 2A). Importantly, by comparing the levels of ChAT protein to HSPs in anti-ChAT co-IP samples, we find enhanced association of P17A/P19A-ChAT with endogenous HSP70 (*p* ≤ 0.001; Figure 2B), HSP90 (*p* ≤ 0.001; Figure 2C) and HSC70 (*p* ≤ 0.001; Figure 2D) relative to wild-type ChAT.

Our initial BioID and co-IP experiments were performed in HEK293 cells as these cells are easily transfected to express heterologous proteins. As our previous studies on ChAT ubiquitination and degradation were carried out with mouse cholinergic SN56 neural cells (Morey et al., 2016), we confirmed our novel results by performing anti-ChAT co-IPs from SN56 cells expressing either wild-type or P17A/P19A-ChAT. Furthermore, to assess whether enhanced association of ChAT with HSC/HSP70 and HSP90 would also be seen in selected CMS-related ChAT mutant proteins, we also evaluated anti-ChAT co-IPs from SN56 cells expressing either V18M- or A513T-ChAT. We chose to investigate these two catalytically-deficient CMS-related mutations as we had shown enhanced ubiquitination and degradation of these ChAT mutant proteins previously (Morey et al., 2016). Importantly, we detected both endogenous HSC70 and HSP90 in anti-ChAT co-IP samples from SN56 cells expressing either wild-type or mutant ChAT proteins (Figure 2E). Unlike HEK293 cells, we did not observe constitutive expression of HSP70 in SN56 cells. By comparing the levels of ChAT proteins to endogenous HSC70 in anti-ChAT co-IP samples, we find enhanced association of P17A/P19A- (*p* ≤ 0.001) and V18M-ChAT (*p* ≤ 0.05), but not A513T-ChAT, with HSC70 compared to wild-type ChAT (Figure 2F). Interestingly, there were no significant differences in association of mutant ChAT with HSP90 in SN56 cells, but there was a trend toward enhanced HSP90 association with P17A/P17A-ChAT (*p* = 0.09; Figure 2G).

To confirm whether HSC70 and HSP90 interact with ChAT *in situ*, we performed a proximity ligation assay (PLA) in SN56 cells expressing wild-type ChAT. As predicted, we observed interaction of wild-type ChAT with endogenous HSC70 and HSP90 in cells (Figure 2H). Confocal images show that the ChAT-HSP PLA complexes are predominantly cytoplasmic in agreement with the reported subcellular distribution of 69-kDa ChAT (Resendes et al., 1999). As controls, PLA complexes were not observed in cells that were transfected with empty vector and incubated with primary antibodies, or when primary antibodies recognizing ChAT, HSC70 or HSP90 were omitted from the assay.

### Inhibition of HSC/HSP70 interaction with client proteins promotes accumulation of high molecular mass triton-insoluble ChAT aggregates

**Figure 3:**
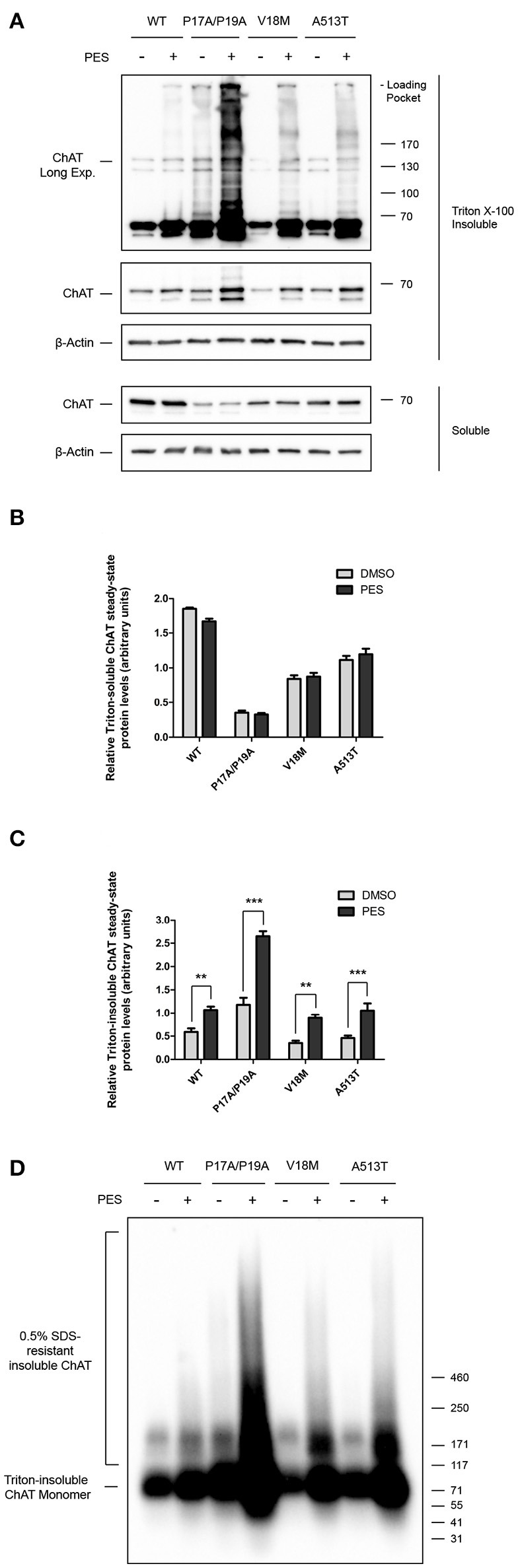
Inhibition of HSC/HSP70 interaction with client proteins promotes accumulation of high molecular mass Triton-insoluble ChAT aggregates. **(A)** Immunoblots from SN56 cells expressing either wild-type or mutant ChAT that were treated for 24 h with either 20 μM PES, a HSC/HSP70 inhibitor, or DMSO-vehicle. Cells were lysed in 0.1% Triton X-100 and fractionated into soluble and insoluble proteins. Triton X-100 insoluble proteins were solubilized prior to SDS-PAGE by denaturing in 2x Laemmli sample buffer with 5% 2-mercaptoethanol. PES treatment resulted in production of high molecular mass Triton-insoluble ChAT proteins that accumulated in the loading pockets of the SDS-PAGE gels (top panel, anti-ChAT long-exposure). The abundance of insoluble ChAT in the loading pockets appears to be greater for mutant ChAT compared to wild-type ChAT, with levels of Triton-insoluble P17A/P19A-ChAT appearing the greatest. **(B)** Treatment with 20 μM PES had no effect on the total levels of Triton-soluble ChAT protein. **(C)** PES treatment significantly increased the levels of Triton-insoluble wild-type (^**^p ≤ 0.01), P17A/P19A- (^***^p ≤ 0.001), V18M- (^**^p ≤ 0.01) and A513T-ChAT (^***^p ≤ 0.001) proteins compared to vehicle-control (two-way ANOVA with Bonferroni's *post-hoc* test, mean ± SEM, *n* = 6). **(D)** Triton-insoluble protein samples from **(A)** were analyzed by semi-denaturing detergent agarose gel electrophoresis (SDD-AGE) revealing the presence of insoluble SDS-resistant ChAT aggregates in PES-treated cells. The abundance of these SDS-resistant ChAT aggregates is greater for mutant ChAT compared to wild-type ChAT, with P17A/P19A-ChAT being the greatest (*n* = 6).

Functionally, HSPs aid in the stabilization of cellular proteins by interacting with exposed peptide sequences rich in hydrophobic amino acids, thereby preventing unintended protein aggregation and promoting protein folding and solubility (McClellan and Frydman, 2001; Xu et al., 2005; Richter et al., 2010). Therefore, to test whether HSC/HSP70 may participate in the folding of ChAT protein, we treated ChAT-expressing SN56 cells with the HSC/HSP70 inhibitor 2-phenylethynesulfonamide (PES) (Figure 3), a small-molecule that interferes with HSC/HSP70-client interactions leading to accumulation and insolubilization of misfolded and aggregation-prone proteins (Leu et al., 2009, 2011). Cells were treated with 20 μM PES for 24 h, then Triton-soluble and insoluble ChAT levels were assessed by immunoblotting to determine levels of misfolded and/or aggregated ChAT protein (Figure 3A). Importantly, PES treatment of cells did not alter the levels of Triton-soluble wild-type or mutant ChAT proteins (Figure 3B), but levels of Triton-insoluble wild-type (*p* ≤ 0.01), P17A/P19A- (*p* ≤ 0.001), V18M- (*p* ≤ 0.01), and A513T-ChAT proteins (*p* ≤ 0.001) were significantly increased compared to levels found in DMSO-treated control cells (Figure 3C). Moreover, immunoblots of Triton-insoluble samples from PES-treated cells revealed accumulation of anti-ChAT immunopositive high molecular mass proteins and the presence of insoluble ChAT that did not enter the stacking gels of the SDS-PAGE gels (Figure 3A, top panel, anti-ChAT immunoblot, long-exposure). The accumulation of high molecular mass Triton-insoluble ChAT proteins is greater for mutant ChAT than for wild-type ChAT, with levels of Triton-insoluble P17A/P19A-ChAT being the greatest. Interestingly, high molecular mass Triton-insoluble P17A/P19A-ChAT was also observed in DMSO-treated cells, though at a lower abundance than in PES-treated cells.

To assess whether the high molecular mass Triton-insoluble ChAT proteins observed following PES treatment may consist of detergent-resistant ChAT aggregates, we performed semi-denaturing detergent agarose gel electrophoresis (SDD-AGE) (Halfmann and Lindquist, 2008) on Triton-insoluble protein samples from Figure 3A. Using anti-ChAT immunoblotting, we observed accumulation of high molecular mass SDS-resistant ChAT protein aggregates from lysates of PES-treated cells, but generally not from DMSO-treated control cells (Figure 3D). The abundance of these insoluble SDS-resistant ChAT aggregates is greater for mutant ChAT compared to wild-type ChAT, with P17A/P19A-ChAT being the greatest. Moreover, insoluble SDS-resistant aggregates of P17A/P19A-ChAT were found in DMSO-treated cells, suggesting that this mutation may promote ChAT insolubility and aggregation under basal conditions. Taken together, these results suggest that folding and solubility of ChAT protein is promoted by interaction of ChAT with HSC/HSP70, and that the catalytically-deficient ChAT mutant proteins tested appear more sensitive to PES-induced insolubilization.

### Inhibition of HSC/HSP70 ATPase activity reduces ChAT steady-state protein levels

**Figure 4:**
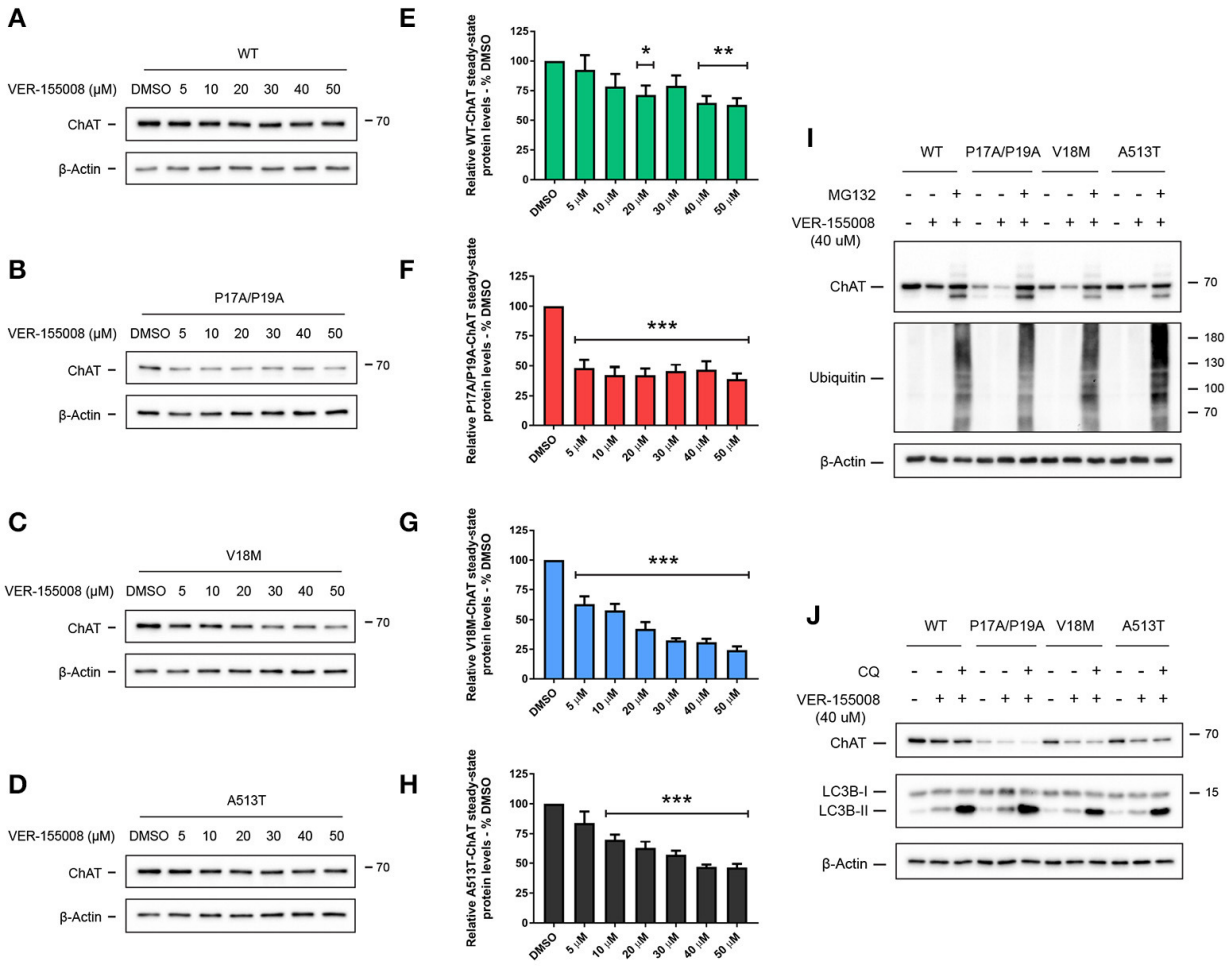
Inhibition of HSC/HSP70 ATPase activity reduces ChAT steady-state protein levels in a dose-related and proteasome-dependent manner. Immunoblots from SN56 cells expressing wild-type **(A)**, P17A/P19A- **(B)**, V18M- **(C)**, or A513T-ChAT **(D)** that were treated for 24 h with 5–50 μM VER-155008, an inhibitor of HSC/HSP70 activity, or with DMSO-vehicle. Steady-state protein levels of wild-type **(E)**, P17A/P19A- **(F)**, V18M- **(G)**, and A513T-ChAT **(H)** are reduced following treatment with VER-155008 as compared to vehicle-control (^**^p ≤ 0.01, ^***^p ≤ 0.001; one-way ANOVA with Dunnett's *post-hoc* test, mean ± SEM, *n* = 4). Mutant ChAT appears more sensitive to HSC/HSP70 inhibition where treatment with 50 μM VER-155008 reduced ChAT steady-state proteins levels, compared to vehicle-control, as follows; wild-type ChAT (38.63% ± 4.97), P17A/P19A-ChAT (61.04% ± 4.17), V18M-ChAT (75.71% ± 2.82), and A513T-ChAT (53.60% ± 2.69). **(I)** Proteasome inhibition by co-treatment with 5 μM MG132 for 18 h attenuates the effects of inhibition of HSC/HSP70 (40 μM VER-155008, 24 h) on ChAT steady-state protein levels in SN56 cells. Control cells were treated with DMSO-vehicle. Proteasome inhibition was validated by immunoblotting for the accumulation of ubiquitinated cellular proteins (*n* = 4). **(J)** Lysosomal inhibition by co-treatment with 50 μM chloroquine (CQ) for 18 h did not prevent the effects of VER-155008 treatment (40 μM, 24 h) on steady-state levels of ChAT protein in SN56 cells. Lysosomal inhibition was validated by immunoblotting for the accumulation of the lysosome-associated protein LC3B-II (*n* = 3).

In addition to client protein interaction, the protein-chaperoning function of HSC/HSP70 requires activity of its N-terminal ATPase domain, and inhibition of this domain by the small-molecule VER-155008 promotes the degradation of HSC/HSP70 clients (Massey et al., 2010; Schlecht et al., 2013). Thus, to determine if HSC/HSP70 activity is required for ChAT protein stability, SN56 cells expressing either wild-type (Figure 4A), P17A/P19A- (Figure 4B), V18M- (Figure 4C) or A513T-ChAT (Figure 4D) were treated with varying concentrations (5–50 μM) of VER-155008 for 24 h. When analyzed by anti-ChAT immunoblotting, we observed dose-related reductions in the steady-state protein levels for both wild-type and mutant ChAT in VER-155008 treated cells. Interestingly, mutant ChAT appears more sensitive than wild-type ChAT to HSC/HSP70 inhibition. A significant decrease in wild-type ChAT steady-state protein level was observed when cells were treated with at least 20 μM VER-155008 (*p* ≤ 0.05; Figure 4E), while lower concentrations of VER-155008 were sufficient to significantly reduce protein levels of P17A/P19A- (5 μM; *p* ≤ 0.001; Figure 4F), V18M- (5 μM; *p* ≤ 0.001; Figure 4G) or A513T-ChAT (10 μM; *p* ≤ 0.001; Figure 4H) compared to DMSO-treated control cells. Furthermore, treatment of ChAT-expressing cells with 50 μM VER-155008 reduced ChAT steady-state protein levels compared to DMSO-treated control cells as follows: wild-type ChAT (38.6 ± 4.97%), P17A/P19A-ChAT (61.0 ± 4.17%), V18M-ChAT (75.7 ± 2.82%) and A513T-ChAT (53.6 ± 2.69%). To test whether the loss of ChAT protein following inhibition of HSC/HSP70 was due to proteasomal degradation of ChAT, we co-treated ChAT-expressing SN56 cells with VER-155008 and the proteasome inhibitor MG132. We observed that treatment of cells expressing either wild-type and mutant ChAT with 40 μM VER-155008 reduced ChAT steady-state protein levels, and that co-treatment of cells with 5 μM MG132 attenuated this effect (Figure 4I). Furthermore, co-treatment of ChAT-expressing SN56 cells with 50 μM chloroquine to inhibit lysosome function (Seglen et al., 1979) did not alter the effects of 40 μM VER-155008 on steady-state levels of either wild-type or mutant ChAT proteins (Figure 4J).

### Inhibition of HSP90 ATPase activity specifically reduces steady-state protein levels of mutant ChAT

**Figure 5:**
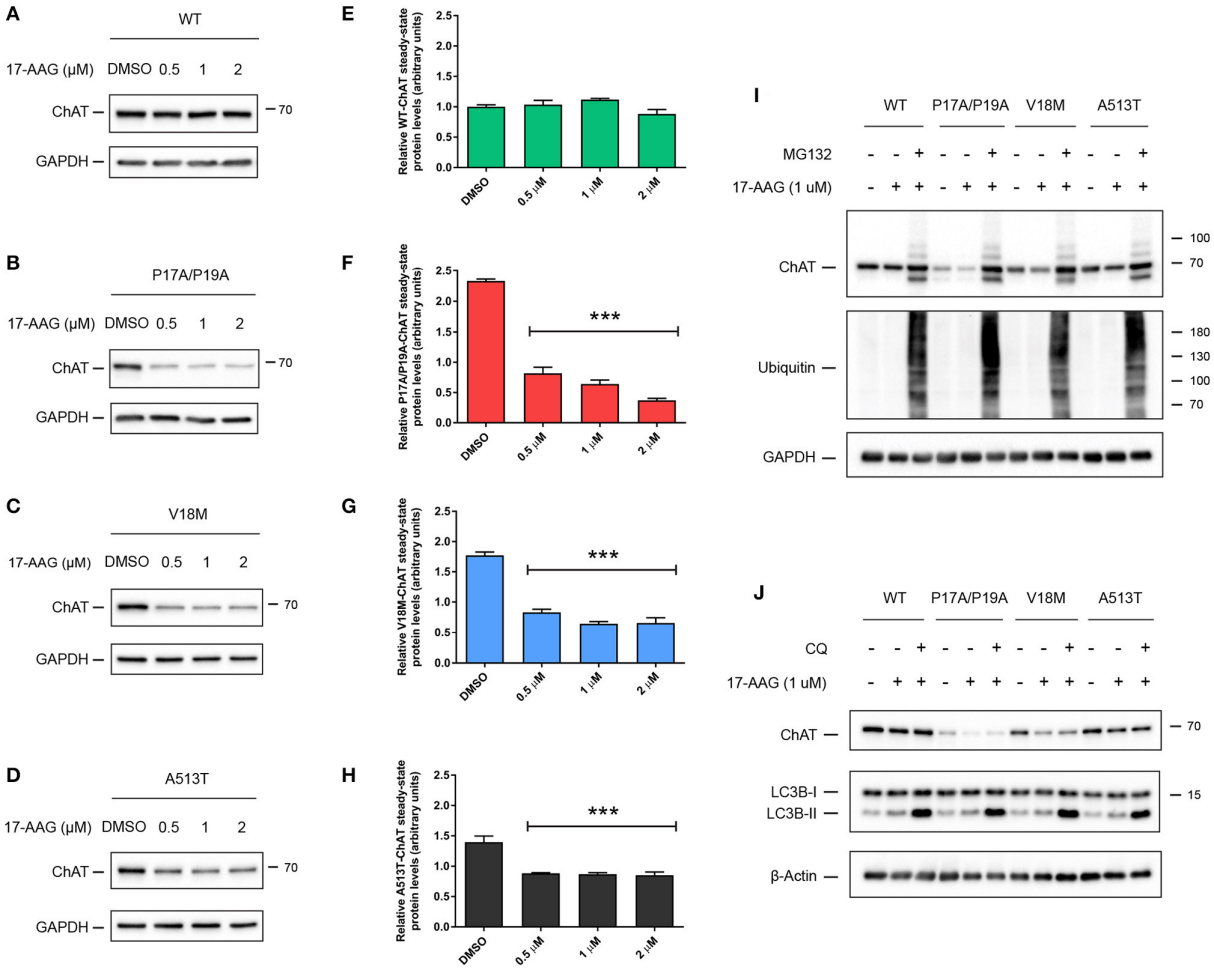
Inhibition of HSP90 ATPase activity specifically reduces steady-state levels of mutant ChAT protein. Immunoblots from SN56 cells expressing wild-type **(A)**, P17A/P19A- **(B)**, V18M- **(C)**, or A513T-ChAT **(D)** that were treated for 24 h with 0.5–2 μM with 17-AAG, an inhibitor of HSP90 activity, or with DMSO-vehicle. **(E)** Treatment of cells with 17-AAG at concentrations up to 2 μM has no effect on the steady-state levels of wild-type ChAT protein. Conversely, steady-state protein levels of P17A/P19A- **(F)**, V18M- **(G)**, and A513T-ChAT **(H)** are reduced following treatment of cells with 17-AAG compared to vehicle-control (^***^p ≤ 0.001; one-way ANOVA with Dunnett's *post-hoc* test, mean ± SEM, *n* = 4). **(I)** Proteasome inhibition by co-treatment with 5 μM MG132 for 18 h attenuates the effects of inhibition of HSP90 (1 μM 17-AAG, 24 h) on ChAT steady-state protein levels in ChAT-expressing SN56 cells. Control cells were treated with DMSO-vehicle. Proteasome inhibition was validated by immunoblotting for the accumulation of ubiquitinated cellular proteins (*n* = 4). **(J)** Lysosomal inhibition by co-treatment with 50 μM chloroquine (CQ) for 18 h did not prevent the effects of 17-AAG treatment (1 μM, 24 h) on steady-state ChAT protein levels in SN56 cells. Lysosomal inhibition was validated by immunoblotting for the accumulation of the lysosome-associated protein LC3B-II (*n* = 3).

Like HSC/HSP70, HSP90 has an N-terminal ATP-binding domain with ATPase activity that is required for HSP90 chaperoning function, and pharmacological inhibition of this domain by the small molecule 17-allylamino-17-demethoxygeldanamycin (17-AAG) promotes proteasomal degradation of HSP90 clients (Krishnamoorthy et al., 2013). To determine if HSP90 activity promotes ChAT stabilization, we treated ChAT-expressing SN56 cells with 0.5–2 μM 17-AAG for 24 h (Figure 5). Analysis by anti-ChAT immunoblotting showed that treatment of cells with concentrations of 17-AAG up to 2 μM had no effect on the steady-state levels of wild-type ChAT protein (Figures 5A,E), but did significantly reduce the steady-state levels of mutant P17A/P19A- (*p* ≤ 0.001; Figures 5B,F), V18M- (*p* ≤ 0.001; Figures 5C,G) and A513T-ChAT proteins (*p* ≤ 0.001; Figures 5D,H) compared to DMSO-treated control cells. To test if the effects of 17-AAG on ChAT protein levels were proteasome-dependent, we co-treated SN56 cells expressing either wild-type or mutant ChAT with 17-AAG and MG132. Treatment of cells with 1 μM 17-AAG alone reduced steady-state protein levels of only mutant ChAT, while co-treatment with 5 μM MG132 attenuated that effect (Figure 5I). Again, lysosomal inhibition by treatment of ChAT-expressing SN56 cells with 50 μM chloroquine did not prevent the effects of 1 μM 17-AAG co-treatment on steady-state levels of either wild-type or mutant ChAT protein (Figure 5J). Overall, these data suggest that while the ATPase activity of HSC/HSP70 is necessary for both wild-type and mutant ChAT protein stability, the ATPase activity of HSP90 is critical for stabilization of mutant ChAT, but not wild-type ChAT protein.

### Inhibition of HSC/HSP70 and HSP90 activity enhances ChAT ubiquitination and reduces ChAT enzymatic activity

**Figure 6:**
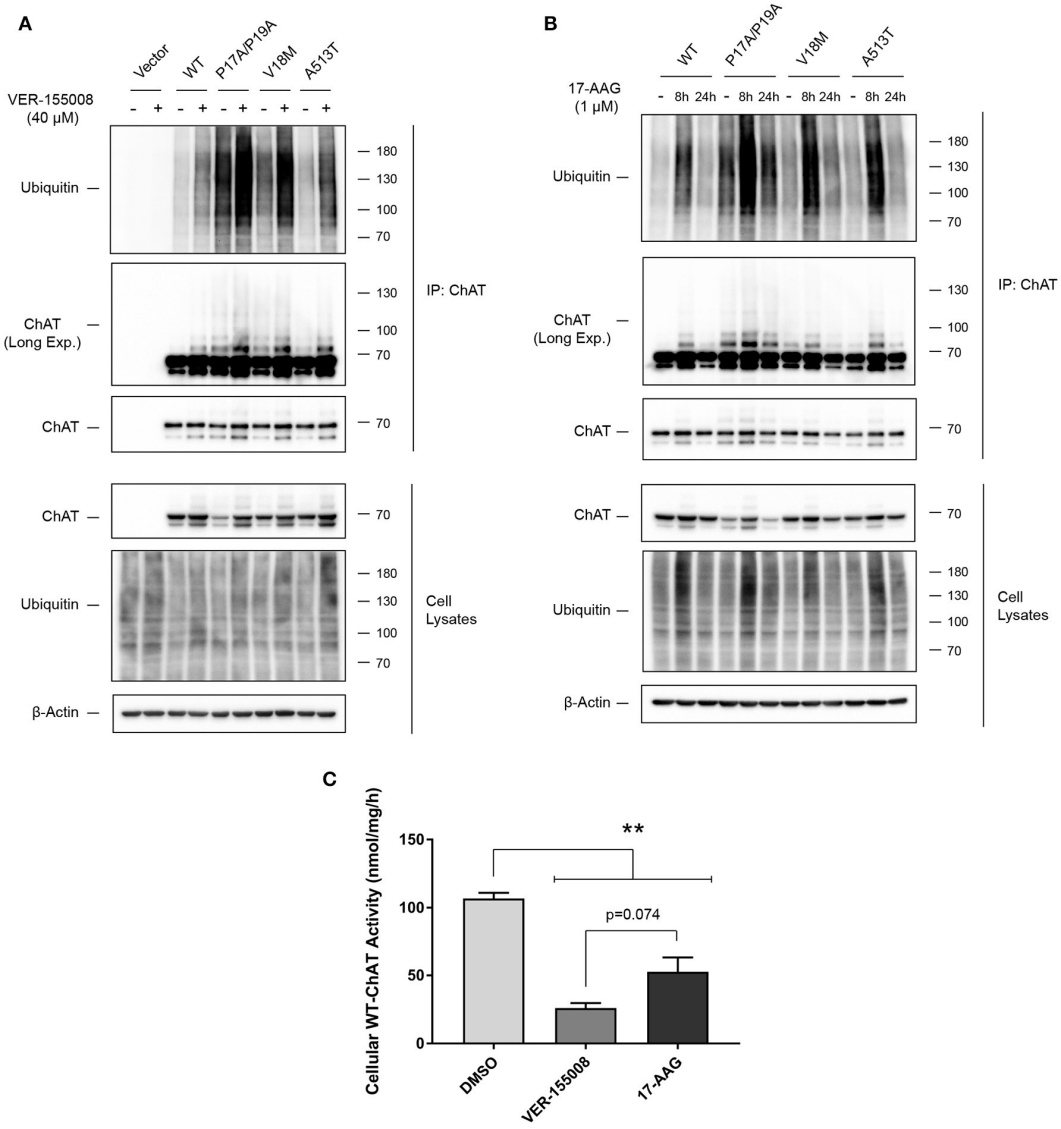
Inhibition of HSC/HSP70 and HSP90 activity enhances ChAT ubiquitination and reduces cellular ChAT enzymatic activity. **(A)** Immunoblots following anti-ChAT IP from ChAT-expressing SN56 cells that were co-treated for 24 h with 40 μM VER-155008 and for the final 6 h with 20 μM MG132 to inhibit degradation of ubiquitinated ChAT. Control cells were transfected with empty vector and/or treated with DMSO-vehicle. Inhibition of HSC/HSP70 by VER-155008 treatment enhanced ChAT ubiquitination, where levels of ubiquitinated mutant ChAT, particularly P17A/P19A-ChAT, are greater than that of wild-type ChAT (*n* = 3). **(B)** Immunoblots following anti-ChAT IP from ChAT-expressing SN56 cells that were co-treated for either 8 or 24 h with 1 μM 17-AAG and for the final 6 h with 20 μM MG132. Inhibition of HSP90 by treatment with 1 μM 17-AAG for 8 h, but not for 24 h, enhanced ChAT ubiquitination where levels of ubiquitinated mutant ChAT, particularly P17A/P19A-ChAT, are greater than that of wild-type ChAT (*n* = 3). **(C)** Cellular activity of wild-type ChAT is reduced following treatment of SN56 cells for 24 h with either 40 μM VER-155008 or 1 μM 17-AAG compared to vehicle-treated (DMSO) cells (^**^p ≤ 0.01). Treatment with VER-155008 trended toward a greater reduction in ChAT activity compared to 17-AAG (*p* = 0.074; one-way ANOVA with Tukey's *post-hoc* test, mean ± SEM, *n* = 3).

In addition to protein folding, HSPs perform essential protein quality control functions by triaging stress-damaged or terminally misfolded proteins for degradation through the ubiquitin-proteasome system (McClellan and Frydman, 2001; Richter et al., 2010). Therefore, to determine if inhibition of either HSC/HSP70 or HSP90 promotes proteasomal degradation of ChAT through enhanced ChAT ubiquitination, we treated ChAT-expressing SN56 cells with either 40 μM VER-155008 for 24 h or 1 μM 17-AAG for either 8 or 24 h, along with 20 μM MG132 during the final 6 h of the incubation to prevent degradation of ubiquitinated ChAT. Using anti-ChAT IP and anti-ubiquitin immunoblotting, we observed that treatment of cells with VER-155008 resulted in enhanced ubiquitination of both wild-type and mutant ChAT compared to DMSO-treated control cells (Figure 6A), with ubiquitination of mutant ChAT, particularly P17A/P19A-ChAT, being greater than that of wild-type ChAT. Surprisingly, treatment of cells with 17-AAG for 24 h had no effect on the ubiquitination of either wild-type or mutant ChAT proteins compared to DMSO-control, but 8 h treatment with 17-AAG did enhance ubiquitination of ChAT with this being greater for mutant ChAT than for wild-type ChAT, particularly for P17A/P19A-ChAT (Figure 6B).

Canonically, ubiquitination targets proteins for proteasomal and/or lysosomal degradation, but it can also regulate subcellular trafficking, receptor endocytosis and enzymatic activity of various proteins (Sadowski et al., 2012). Therefore, to determine if HSP inhibition may also affect ChAT enzymatic activity, we treated SN56 cells expressing heterologous wild-type ChAT with either 40 μM VER-155008 or 1 μM 17-AAG for 24 h, then analyzed cellular ChAT activity using a modified radioenzymatic assay (Fonnum, 1969; Pongrac and Rylett, 1998). Interestingly, we found that enzymatic activity of wild-type ChAT is significantly reduced following treatment of SN56 cells for 24 h with either 40 μM VER-155008 or 1 μM 17-AAG compared to DMSO-treated control cells (p ≤ 0.01; Figure 6C). The reduction in cellular ChAT activity appears greater in cells treated with VER-155008 than with 17-AAG, but this did not achieve statistical significance (*p* = 0.074). Importantly, these data show that while inhibition of HSP90 by 17-AAG treatment had no effect on the steady-state protein levels of wild-type ChAT (Figures 5A,E), this treatment did significantly reduce wild-type ChAT enzyme activity similar to that observed with HSC/HSP70 inhibition.

### ChAT interacts with the HSP-associated E3 ubiquitin ligase C-terminus of HSC70-interaction protein (CHIP)

**Figure 7:**
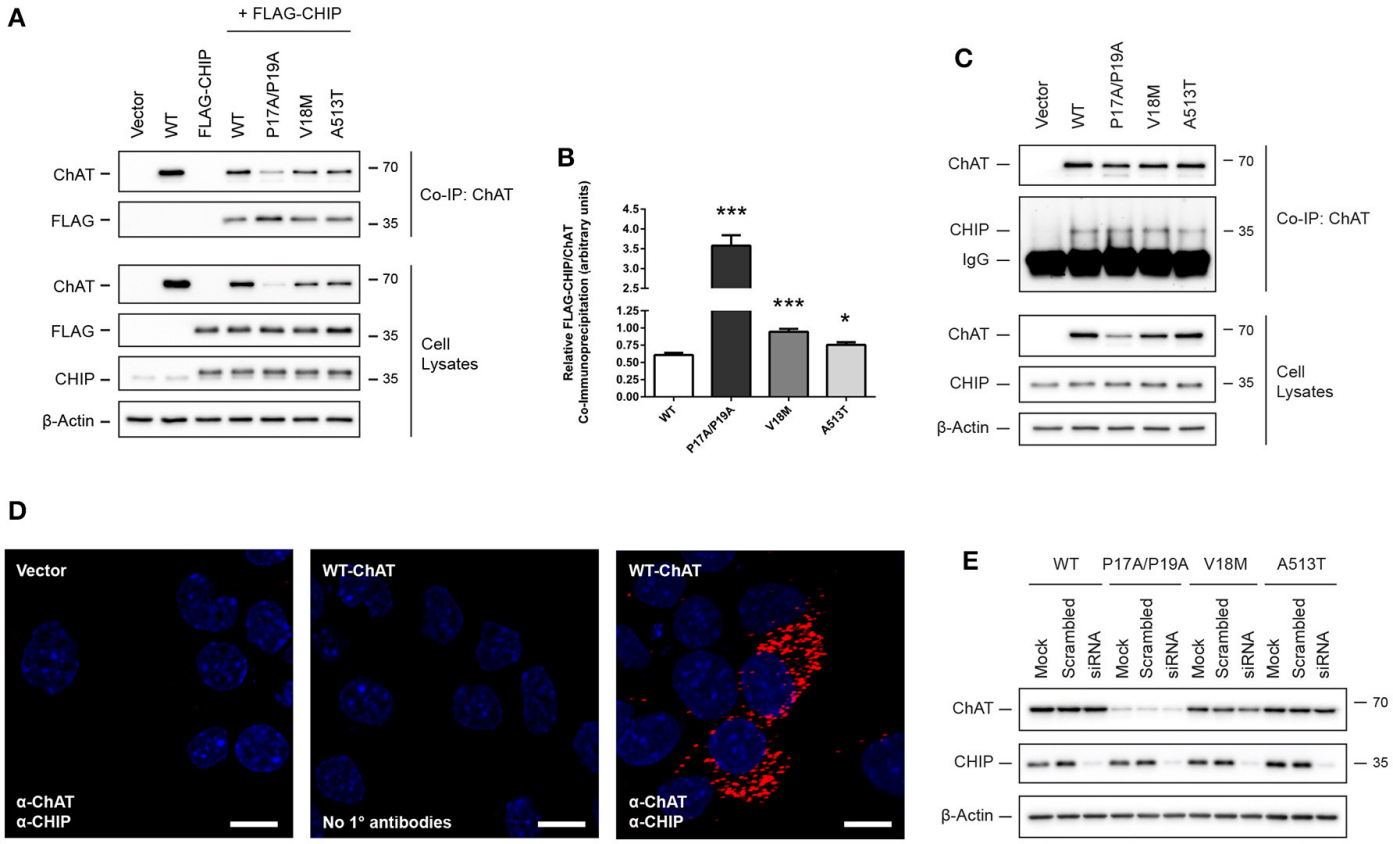
ChAT interacts with the HSP-associated E3 ubiquitin ligase C-terminus of HSC70-interaction protein (CHIP). **(A)** Immunoblots showing co-IP of ChAT with FLAG-CHIP from SN56 cells co-expressing either wild-type or mutant ChAT protein with FLAG-tagged CHIP. Control cells were transfected with either empty vector or to express either wild-type ChAT or FLAG-CHIP alone. **(B)** Co-IP of ChAT with FLAG-CHIP is enhanced for P17A/P19A- (^***^p ≤ 0.001), V18M- (^***^p ≤ 0.001), and A513T-ChAT (^*^p ≤ 0.05) as compared to wild-type ChAT (one-way ANOVA with Dunnett's *post-hoc* test, mean ± SEM, *n* = 5). **(C)** Co-IP of wild-type and mutant ChAT with endogenous CHIP following anti-ChAT co-IP from ChAT-expressing SN56 cells (*n* = 3). **(D)** Detection of *in situ* ChAT interactions with endogenous CHIP by proximity ligation assay (PLA) in SN56 cells expressing wild-type ChAT. Formalin-fixed cells were first co-labeled with goat anti-ChAT together with rabbit anti-CHIP primary antibodies, then incubated with oligonucleotide-linked secondary antibodies. Following DNA ligation and DNA amplification using the Duolink *In Situ* Orange Kit (Sigma), *in situ* ChAT-CHIP interactions were imaged by confocal microscopy. Positive *in situ* ChAT-CHIP interactions were visualized as fluorescent red dots while nuclei were stained with DAPI (blue). Control cells were either transfected with empty vector or primary antibodies omitted from the assay (No 1° antibodies). Images are representative of 4 independent experiments; scale bars are 10 μm. **(E)** siRNA-mediated knock-down of CHIP has no effect on the steady-state protein levels of either wild-type or mutant ChAT. ChAT-expressing SN56 cells were co-transfected with 25 nM of either anti-CHIP siRNA or scramble-control siRNA for 72 h. Control cells were mock-transfected (*n* = 4).

The E3 ubiquitin ligase CHIP/Stub1 ubiquitinates proteins in an HSP-dependent manner through direct interaction with either HSC/HSP70 or HSP90 and their bound client proteins (Ballinger et al., 1999; Connell et al., 2001; Zhang et al., 2015). To determine if ChAT interacts with CHIP, we performed ChAT co-IPs from SN56 cells co-expressing either wild-type or mutant ChAT proteins with FLAG-tagged CHIP (Figure 7A). FLAG-CHIP was detected in anti-ChAT co-IP samples for both wild-type and mutant ChAT on anti-FLAG immunoblots. By comparing the levels of FLAG-CHIP to ChAT protein, we observed enhanced co-IP of FLAG-CHIP with P17A/P19A- (*p* ≤ 0.001), V18M- (*p* ≤ 0.001), and A513T-ChAT (*p* ≤ 0.05) compared to wild-type ChAT (Figure 7B). Association of wild-type and mutant ChAT with endogenous CHIP was confirmed by anti-ChAT co-IP from ChAT-expressing SN56 cells with follow-up anti-CHIP immunoblotting (Figure 7C). We also performed PLA experiments to visualize and confirm, by confocal microscopy, the presence of *in situ* interactions between wild-type ChAT and endogenous CHIP in SN56 cells (Figure 7D). We did not observe *in situ* interactions in control cells that were either transfected with empty vector or when primary antibodies toward ChAT or CHIP were omitted from the assay. To determine if ChAT is a substrate for CHIP-mediated degradation, we tested whether overexpression of FLAG-CHIP promotes the degradation of ChAT and, conversely, if siRNA-mediated knock-down of endogenous CHIP prevents ChAT degradation in SN56 cells. Interestingly, while overexpression of FLAG-CHIP led to enhanced proteasomal degradation of both wild-type and mutant ChAT (Supplementary Figure S5), siRNA-mediated knock-down of endogenous CHIP had no effect on the steady-state levels of either wild-type or mutant ChAT proteins as assessed by anti-ChAT immunoblotting (Figure 7E). Thus, while these results identify CHIP as a novel ChAT protein-interactor in cells, the function/s for this interaction is not known.

### Inhibition of the co-chaperone Cdc48/p97/Valosin-containing protein (VCP) prevents degradation of ubiquitinated ChAT

**Figure 8:**
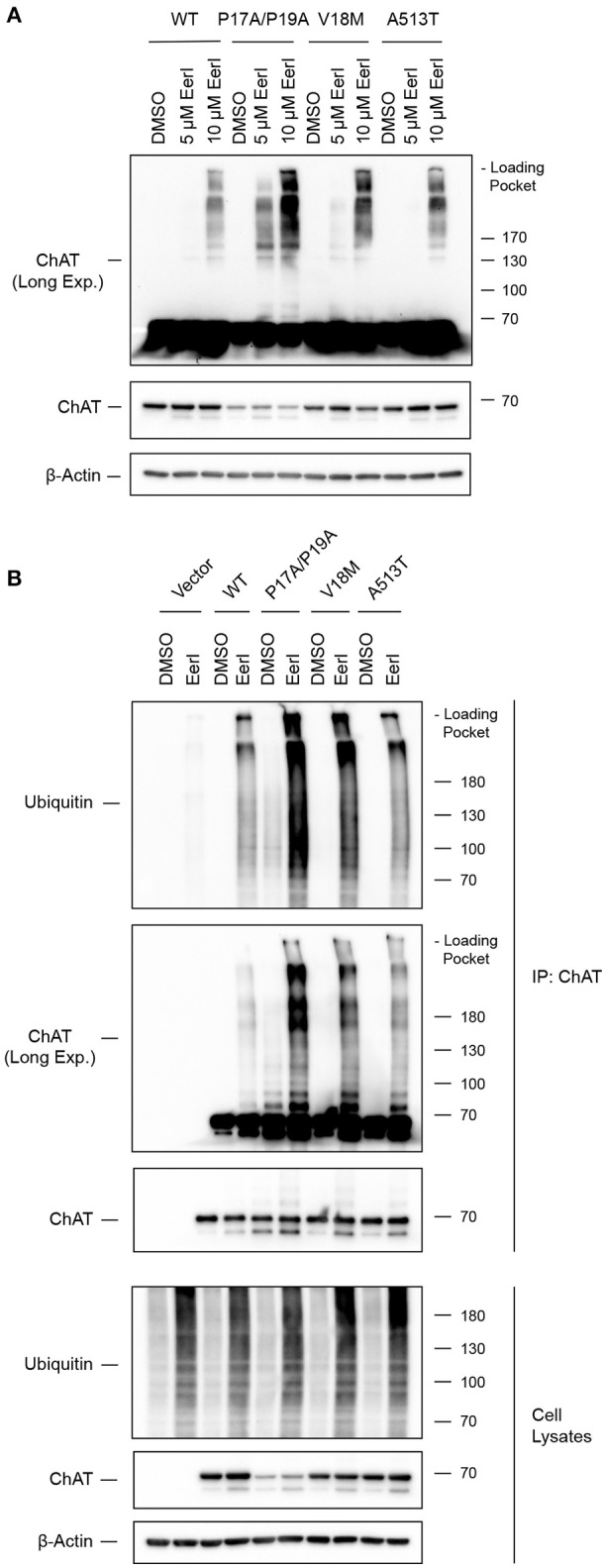
Inhibition of the co-chaperone Cdc48/p97/Valosin-containing protein (VCP) prevents degradation of ubiquitinated ChAT. **(A)** Immunoblots from ChAT-expressing SN56 cells that were treated for 18 h with 5–10 μM Eeyarestatin-I (Eer1), a VCP inhibitor, or with DMSO-vehicle. Inhibition of VCP with 10 μM Eer1 resulted in the accumulation of high molecular mass ChAT-immunopositive proteins and the presence of insoluble ChAT in the loading pockets of the SDS-PAGE gels (top panel, anti-ChAT long-exposure). The accumulation of high molecular mass ChAT appears greater for mutant ChAT compared to wild-type ChAT, with levels of P17A/P19A-ChAT being the greatest (*n* = 3). **(B)** Inhibition of VCP attenuates the degradation of ubiquitinated ChAT. Ubiquitinated ChAT was analyzed by anti-ubiquitin immunoblotting following anti-ChAT IP from ChAT-expressing SN56 cells that were treated for 18 h with 10 μM Eer1. Control cells were transfected with empty vector and/or treated with DMSO-vehicle. The abundance of ubiquitinated ChAT following VCP inhibition is greater for mutant ChAT, particularly P17A/P19A-ChAT, compared to wild-type ChAT (*n* = 3).

The co-chaperone VCP modulates HSP clients through interaction with HSC/HSP70 (Pleasure et al., 1993) and HSP90 (Prince et al., 2005). Previously, we showed that VCP interacts with ChAT in IMR32 neural cells exposed to oligomeric Aβ_1−42_ (Dobransky et al., 2003). Therefore, to examine if wild-type and/or mutant ChAT protein stability is regulated by VCP under basal conditions, we treated ChAT-expressing SN56 cells for 18 h with 5–10 μM Eeyarestatin-I (Eer1), a small-molecule VCP inhibitor (Wang et al., 2008, 2010). By anti-ChAT immunoblotting, we observed accumulation of high molecular mass wild-type and mutant ChAT-immunopositive proteins following cell treatment with 10 μM Eer1, but not in control cells treated with DMSO (Figure 8A). The abundance of high molecular mass ChAT is greater for mutant ChAT proteins than wild-type ChAT, with levels of P17A/P19A-ChAT being the greatest. Additionally, Eer1 treatment led to accumulation of insoluble high molecular mass ChAT proteins in the loading pockets of the SDS-PAGE gels.

VCP has been shown previously to promote proteasome-dependent degradation of ubiquitinated proteins from both the ER and the cytoplasm (Dai and Li, 2001; Meyer et al., 2012). Furthermore, VCP inhibition by Eer1 treatment can result in the accumulation of ubiquitinated VCP substrates by preventing their degradation (Wang et al., 2008, 2010). Therefore, to determine if high molecular mass ChAT observed following Eer1 treatment represents ubiquitinated ChAT, and therefore whether VCP inhibition may prevent degradation of ubiquitinated ChAT, we completed anti-ChAT IPs from ChAT-expressing SN56 cells treated with 10 μM Eer1 for 18 h. Importantly, to maintain proteasome function and to allow for degradation of ubiquitinated ChAT in cells treated with DMSO-control we did not co-treat cells with MG132. Following anti-ubiquitin immunoblotting of anti-ChAT IP samples, we confirmed that VCP inhibition prevented the degradation of ubiquitinated ChAT compared to DMSO-treated cells (Figure 8B). The abundance of ubiquitinated ChAT following VCP inhibition is greater for mutant ChAT compared to wild-type ChAT, with levels of ubiquitinated P17A/P19A-ChAT being the greatest. These results suggest that the co-chaperone VCP has a role in regulating the degradation of ubiquitinated ChAT, a mechanism that is shared between both wild-type and mutant ChAT.

## Discussion

In the current study, we identified novel mechanisms for the regulation of ChAT protein stability involving multiple molecular chaperones. In brief, we show that ChAT interacts with and is a client for the heat shock proteins HSC/HSP70 and HSP90, where inhibition of HSC/HSP70 protein interactions results in accumulation of insoluble ChAT protein, and inhibition of HSC/HSP70 and HSP90 activity led to reduced cellular ChAT activity, along with enhanced ChAT ubiquitination and proteasome-dependent loss of ChAT protein. The effects of HSP inhibition appear greater for mutant ChAT (P17A/P19A-, V18M-, and A513T-ChAT) than for wild-type ChAT. Though we show that ChAT interacts with the HSP-associated E3 ubiquitin ligase CHIP, siRNA-mediated knock-down of CHIP had no effect on ChAT protein levels. Lastly, we show that the ER- and HSP-associated co-chaperone VCP regulates the degradation of ubiquitinated ChAT.

Canonically, HSPs such as HSC/HSP70 and HSP90 have been characterized for their role in the folding and stabilization of nascent proteins, as well as refolding of aggregation- and misfolding-prone proteins during proteotoxic stress (Mosser et al., 1997; McClellan and Frydman, 2001; Richter et al., 2010). Multiple HSPs can act together to chaperone client proteins in a cooperative and processive manner, with initial recognition and folding of proteins completed by an HSP40-HSC/HSP70 complex, followed by transfer to HSP90 by HSP70-HSP90 organizing protein (Hop) and subsequent final folding by HSP90 (Wegele et al., 2004; Alvira et al., 2014). In the present study, inhibition of HSC/HSP70 led to the destabilization and degradation of both wild-type and mutant ChAT, while inhibition of HSP90 reduced only cellular ChAT activity with no effect on wild-type ChAT steady-state protein levels. Thus, HSP90 acts downstream of HSC/HSP70 to interact with semi- or pre-folded proteins where formation of stable HSP90-client interactions is promoted by availability of labile solvent-exposed hydrophobic domains, such as those found in ligand-binding domains and enzyme active sites (Xu et al., 2005; Pratt et al., 2008). As such, HSP90 plays an essential role in the structural maturation and conformational activation of various cellular kinases or enzymes involved in a wide array of cell signaling pathways, such as endothelial nitric oxide synthase (eNOS) (Takahashi and Mendelsohn, 2003) and the receptor tyrosine kinase ErbB2 (Xu et al., 2001). This contrasts with HSC/HSP70 that interacts with a diverse and extensive range of nascent non-native proteins (20% of all nascent proteins) that minimally contain solvent-exposed regions enriched with 4–7 hydrophobic residues (Fourie et al., 1994; Rudiger et al., 1997; Thulasiraman et al., 1999). Thus, we propose that ChAT protein folding, stability and cellular activity is regulated in a similar processive manner by HSC/HSP70 and HSP90 respectively. It is important to note that inhibition of either HSC/HSP70 or HSP90 resulted in the loss of cellular ChAT activity, suggesting that cooperation of these HSPs is essential for the formation of natively-folded and active ChAT enzyme, and thus may be critical to cholinergic neural function.

The primary goal of our study was to identify cellular factors that regulate the stability of ChAT protein, particularly mechanisms that may be responsible for the enhanced ubiquitination of mutant ChAT proteins that we observed previously (Morey et al., 2016). While our data suggest a critical role for HSC/HSP70 and HSP90 in the folding and stabilization of wild-type and/or mutant ChAT, these HSPs are also well-known for their ability to triage mutated, misfolded and aggregated proteins for proteolytic degradation through coordination with the HSP-associated E3 ubiquitin ligase CHIP/Stub1 (Ballinger et al., 1999; Connell et al., 2001; Murata et al., 2001). HSP-mediated protein degradation often requires the formation of pro-degradative trimeric complexes between client-bound HSC/HSP70 or HSP90 and the N-terminal tetracopeptide repeat (TPR) domains of CHIP (Ballinger et al., 1999; Smith et al., 2013; Zhang et al., 2015). As such, studies have reported on the regulation of HSC/HSP70 and HSP90 client proteins through CHIP-mediated ubiquitination, including hypoxia-inducible factor 1-alpha (HIF-1α) (Luo et al., 2010) and ErbB2 (Zhou et al., 2003). The E3 ubiquitin ligase/s responsible for the ubiquitination of wild-type and/or mutant ChAT are currently unknown, and while we show here that ChAT interacts with endogenous CHIP *in situ*, siRNA knock-down of CHIP failed to prevent the degradation of ChAT protein. One possible reason for this observation is that other HSP-associated E3 ubiquitin ligases may regulate ChAT ubiquitination as previous studies have demonstrated functional redundancy for CHIP-mediated degradation of HSP clients in *CHIP*-/- mouse fibroblasts (Morishima et al., 2008). Importantly, the E3 ubiquitin ligase Parkin associates with both HSPs and CHIP to co-regulate the chaperone-mediated degradation of HSP clients such as neuronal nitric oxide synthase (nNOS) and various polyglutamine-expanded proteins (Morishima et al., 2008; Kumar et al., 2012). In addition, other cytosolic E3 ubiquitin ligases can also regulate the ubiquitination of nascent and misfolded HSP clients, including E6-associated protein (E6-AP) and the N-end rule E3 ligase Ubr1 (Mishra et al., 2009; Summers et al., 2013). Thus, we hypothesize that alternative HSP-related E3 ubiquitin ligases, such as Parkin, may regulate ChAT ubiquitination, potentially in cooperation with CHIP.

Multiple co-chaperones regulate the function of HSPs, including HSP40, Hop and CHIP. We showed previously that the co-chaperone Cdc48/p97/VCP can interact with ChAT in neural cells exposed to Aβ (Dobransky et al., 2003). VCP is a member of the AAA-ATPase family that has diverse cellular functions, including the ability to interact with and modulate HSC/HSP70 and HSP90 clients (Pleasure et al., 1993; Prince et al., 2005), and interact with ubiquitinated proteins to regulate their subcellular trafficking and degradation (Dai and Li, 2001; Meyer et al., 2012). In the present study, we show that pharmacological inhibition of VCP led to the accumulation of ubiquitinated ChAT and prevented its degradation. Related to its proteolytic functions, studies have demonstrated a role for VCP in the handling and degradation of protein aggregates (Ju et al., 2008), as well as in the degradation of ubiquitinated proteins by the autophagy-lysosomal system (Ju et al., 2009; Tresse et al., 2010). While the autophagy-lysosomal system is one possible mechanism involved in the VCP-mediated degradation of ubiquitinated ChAT, we have shown both previously and within this study that ChAT protein degradation is largely unaffected by lysosomal inhibition and, conversely, is regulated chiefly by the proteasome (Morey et al., 2016). Interestingly, VCP is best known for its role in promoting the proteasome-dependent degradation of ER-associated protein degradation (ERAD) substrates (Dai and Li, 2001; Meyer et al., 2012), where VCP is critical for ER-to-cytoplasm transfer of proteins ubiquitinated in the ER, thereby directly mediating degradation of ERAD substrates by the cytosolic 26S-proteasome (Ye et al., 2005). Furthermore, VCP influences ubiquitination of ERAD substrates by interacting with the ER-associated E3 ubiquitin ligases gp78/AMFR and Hrd1/SYVN1 (Zhong et al., 2004; Ye et al., 2005; Ballar et al., 2011) that act downstream of 78-kDa glucose-regulated protein (GRP-78), an ER-associated HSP known to promote protein folding and stability of nascent proteins in a manner similar to that of HSC/HSP70 (Hirsch et al., 2009). Therefore, an alternative possibility by which VCP regulates the degradation of ubiquitinated ChAT may involve ERAD-associated mechanisms in addition to those related to cytosolic HSPs, and thus we hypothesize that ChAT may be regulated by various cytosolic and/or ER-associated chaperones that act cooperatively to promote ChAT protein function. Importantly, while our data suggest that the proteasomal degradation of ubiquitinated ChAT is promoted by VCP, it is unclear whether this pro-degradative function of VCP is primarily HSP- or ER-associated; this requires further investigation.

We showed previously that ubiquitination of the catalytically-deficient and CMS-related mutant ChAT proteins V18M- and A513T-ChAT, as well as P17A/P19A-ChAT, is enhanced, resulting in increased proteasomal degradation and loss of mutant ChAT protein (Morey et al., 2016). All three of these mutations are positioned distal to the ChAT active site (Kim et al., 2006; Shen et al., 2011), and while the mechanisms responsible for the loss of mutant ChAT protein in mammalian cells are not clear, one possibility is that these mutations may produce local changes to the ChAT protein secondary structure that are responsible for both the impairment of ChAT catalytic activity and that target these ChAT mutants for chaperone-mediated degradation. In the present study, we observed enhanced interaction of mutant ChAT proteins with HSC/HSP70 and HSP90 and increased sensitivity of mutant P17A/P19A-ChAT and CMS-related V18M- and A513T-ChAT to HSP inhibition where, importantly, HSP90 inhibition resulted in the proteasomal loss of mutant but not wild-type ChAT. In general, HSPs aid in the stabilization of their clients through a process of kinetic partitioning, whereby they interact with exposed hydrophobic domains within cellular proteins that is thought to prevent client protein misfolding and aggregation (Rudiger et al., 1997; Xu et al., 2005; Kim et al., 2013). In support of this, we found that inhibition of HSC/HSP70-client interactions by PES treatment resulted in loss of ChAT protein solubility and the generation of detergent-resistant ChAT aggregates; the abundance of these PES-induced insoluble ChAT proteins was greatest for mutant ChAT. Interestingly, insoluble aggregates of mutant P17A/P19A-ChAT protein was observed in cells with functional HSPs, suggesting that the folding of this mutant ChAT protein may be compromised under basal conditions resulting in the production of catalytically-deficient ChAT that may be susceptible to misfolding and inactivation. While our results suggest that HSC/HSP70 and HSP90 are critical for the stabilization of mutant ChAT, these HSPs are also known to promote the degradation of misfolded proteins through the ubiquitin-proteasome system (McClellan and Frydman, 2001; Kim et al., 2013; Zhang et al., 2015). Importantly, we observed enhanced ubiquitination of ChAT following HSP inhibition, suggesting that impairment of HSP-mediated folding of ChAT protein may promote the ubiquitination and degradation of misfolded ChAT. Therefore, taken together, our data suggest that mutations, such as CMS-related V18M and A513T, may compromise ChAT protein folding and lead to enhanced chaperone-mediated ChAT degradation and/or enhanced dependence of mutant ChAT proteins on HSPs to maintain mutant ChAT stability and function.

Our HSP observations related to mutant ChAT are significant as protein misfolding and enhanced functional dependence on molecular chaperones is frequently observed for mutated and disease-relevant proteins (Valastyan and Lindquist, 2014; Hartl, 2017). Thus, these observations may represent a general HSP-related mechanism for the regulation of ChAT protein stability that may apply to not only various CMS-related ChAT mutants beyond those studied presently, but also to the maintenance of ChAT function during cellular stress and disease. Alterations in the catalytic activity of ChAT have been observed in several protein misfolding neurological diseases, including AD and Huntington's disease (Lange et al., 1992; Pakaski and Kalman, 2008), both of which are known to exhibit dysfunction of HSPs and other molecular chaperones, accumulation of aggregate-prone proteins, and build-up of proteotoxic and oxidative stress (Mati et al., 2014; Lackie et al., 2017). Protein oxidation with subsequent formation of aberrant stress-induced disulfide bonds during cellular exposure to oxidative stress can result in misfolding of cellular proteins with severe functional consequences (Cumming et al., 2004). Interestingly, ChAT has a higher-than-average cysteine content (3.2% vs. 1.6% for intracellular proteins) (Fahey et al., 1977) which may sensitize ChAT to oxidative damage. In support, Nunes-Tavares et al. (2012) demonstrated that cellular ChAT activity is reduced following exposure of neural cells to oligomeric Aβ_1−42_, a molecular hallmark of AD and stimulator of cellular oxidative stress (Butterfield, 2002; Pakaski and Kalman, 2008). Additionally, our lab has shown that Aβ_1−42_ treatment results in stable interaction of ChAT with VCP (Dobransky et al., 2003) and in the current study we provide evidence that VCP may regulate and promote degradation of ubiquitinated ChAT, suggesting that ChAT ubiquitination may be altered during cellular stress. The E3 ligase Parkin is another example of a cysteine-rich intracellular protein (7.5%) that has been shown to misfold, inactivate and aggregate following exposure to H_2_O_2_ (Meng et al., 2011). Therefore, it will be important to investigate if ChAT protein folding and stability is altered during cellular stress and disease, and whether HSPs play a protective role to maintain ChAT function under these stresses.

HSPs and other molecular co-chaperones are best characterized for their essential roles in the folding of nascent and misfolded proteins. In this study, we provide evidence for the regulation of ChAT protein stability by a network of cytoplasmic and ER-associated chaperones that includes HSC/HSP70, HSP90 and VCP. Furthermore, regulation of ChAT mutants by these chaperones is altered, suggesting a mechanism that may be relevant to other CMS-related ChAT mutant proteins or to ChAT during cellular stress and disease. Taken together, our results support further research into the roles of HSPs and VCP on ChAT function under both physiological conditions and during cellular stress and disease.

## Author contributions

TM and RR designed experiments and performed data analysis. TM completed experimental work. WW-N and CS assisted with PLA experiments and confocal imaging. All authors contributed to writing this manuscript through editing and revisions.

### Conflict of interest statement

The authors declare that the research was conducted in the absence of any commercial or financial relationships that could be construed as a potential conflict of interest.

## Abbreviations

17-AAG: 17-allylamino-17-demethoxygeldanamycin
Aβ: β-amyloid
ACh: acetylcholine
AD: Alzheimer's disease
BioID: proximity-dependent biotin identification
ChAT: choline acetyltransferase
CHIP: C-terminus of HSC70-interacting protein
CMS: congenital myasthenic syndrome
CQ: chloroquine
CTab: anti-ChAT carboxyl-terminal peptide antibody
E6-AP: E6-associated protein
Eer1: Eeyarestatin I
eNOS: endothelial nitric oxide synthase
ER: endoplasmic reticulum
ERAD: endoplasmic-reticulum-associated protein degradation
GRP-78: 78-kDa glucose-regulated protein
HIF-1α: hypoxia-inducible factor 1-alpha
Hop: HSP70-HSP90 organizing protein
HSC70: heat shock cognate 71 kDa protein
HSP: heat shock protein
IP: immunoprecipitation
LC-ESI-MS/MS: liquid chromatography-electrospray ionization-tandem mass spectrometry
MALDI-TOF-MS: matrix-assisted laser desorption/ionization time-of-flight mass spectrometry
NLS: nuclear localization signal
nNOS: neuronal nitric oxide synthase
PD: pull-down
PES: 2-phenylethynesulfonamide
PLA: proximity ligation assay
RIPA: radioimmune precipitation buffer
SDD-AGE: semi-denaturing detergent agarose gel electrophoresis
SH3: Src-homology 3
VCP: Valosin-containing protein.
