# Supplementary figures and images for: Chaperone-Mediated Regulation of Choline Acetyltransferase Protein Stability and Activity by HSC/HSP70, HSP90, and p97/VCP
Source: Front Mol Neurosci. 2017 Dec 12;10:415. doi: 10.3389/fnmol.2017.00415 (PMC5733026; doi:10.3389/fnmol.2017.00415)

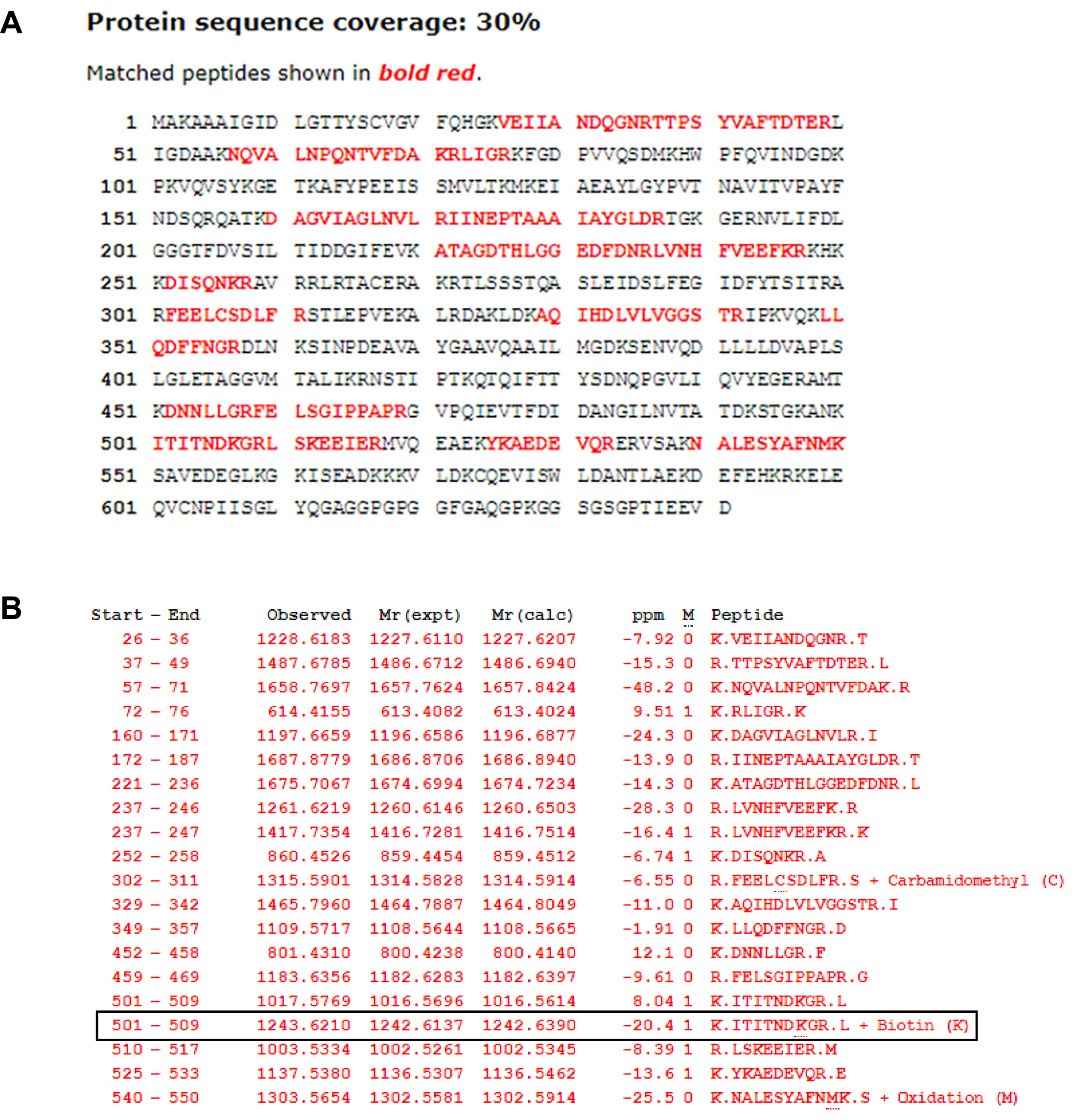

Supplement: Supplementary file 1 [file Image1.JPEG]

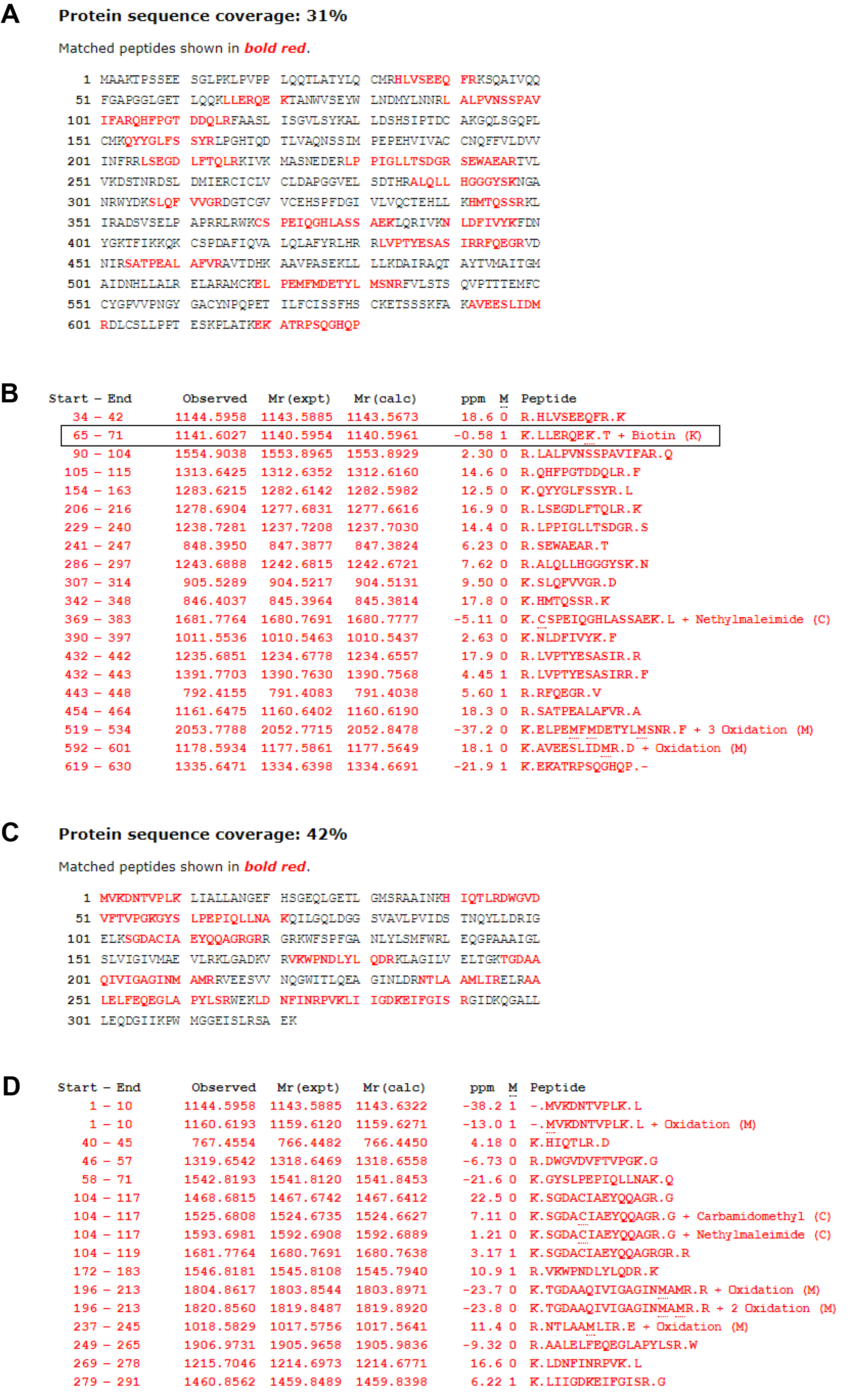

Supplement: Supplementary file 2 [file Image2.JPEG]

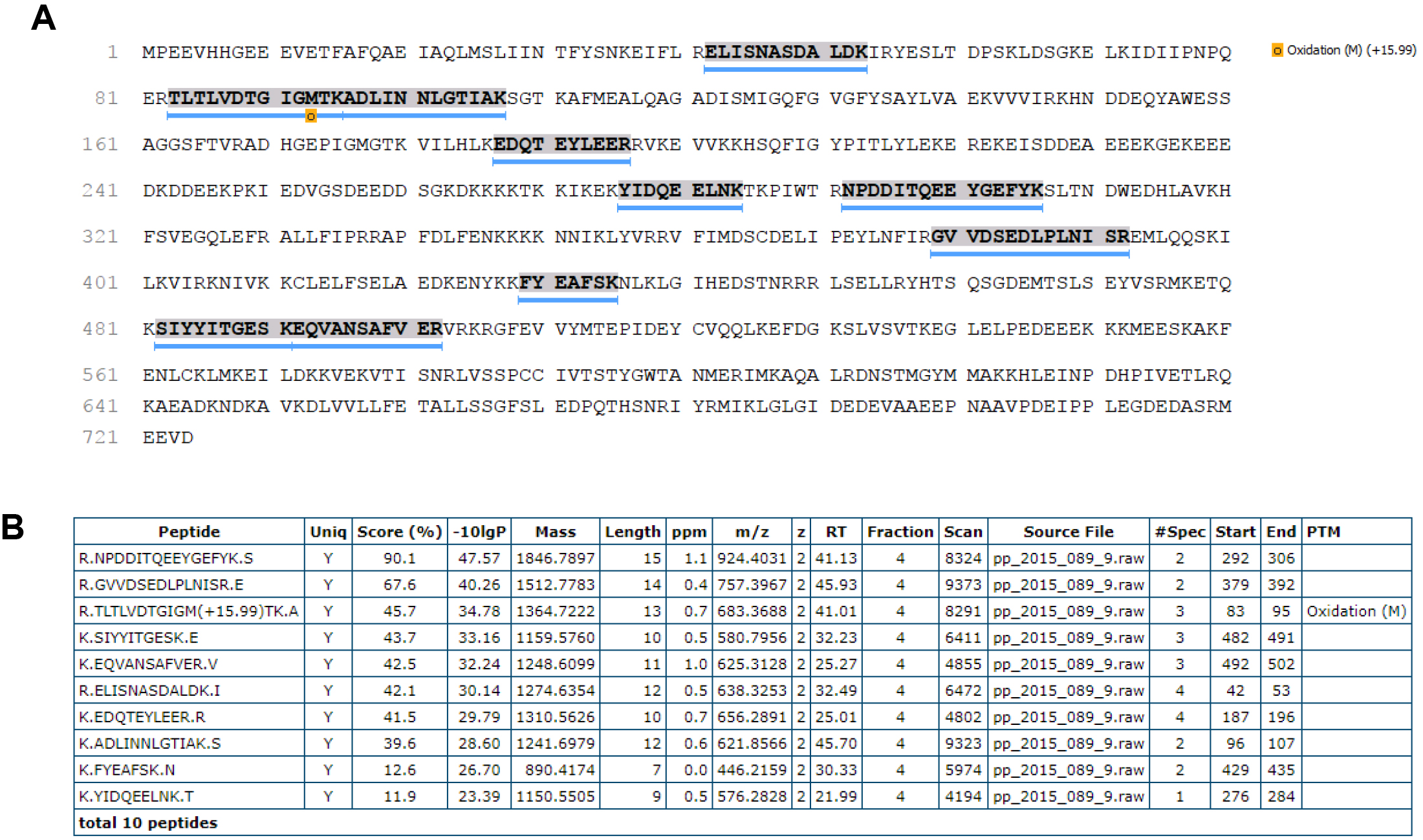

Supplement: Supplementary file 3 [file Image3.JPEG]

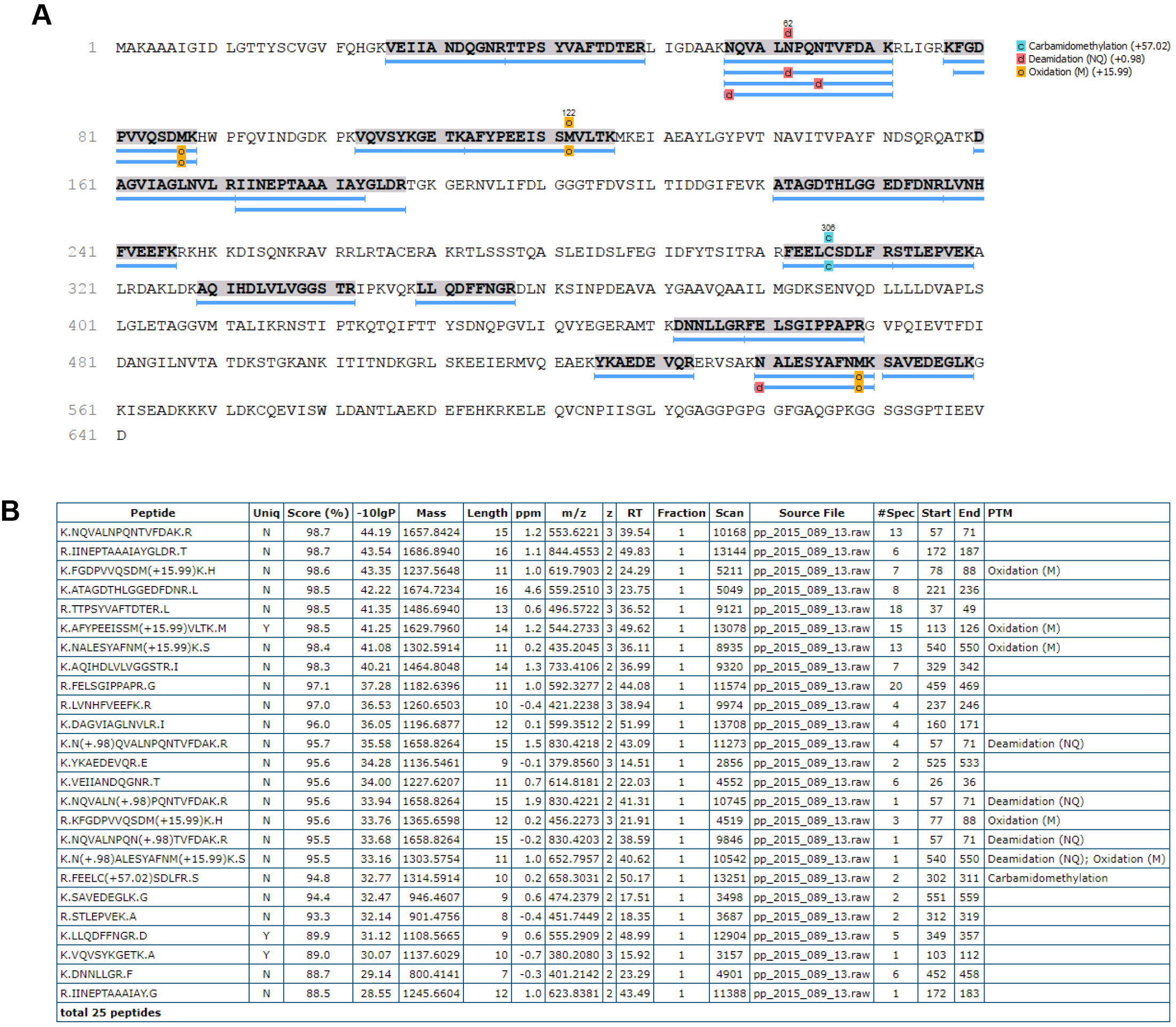

Supplement: Supplementary file 4 [file Image4.JPEG]

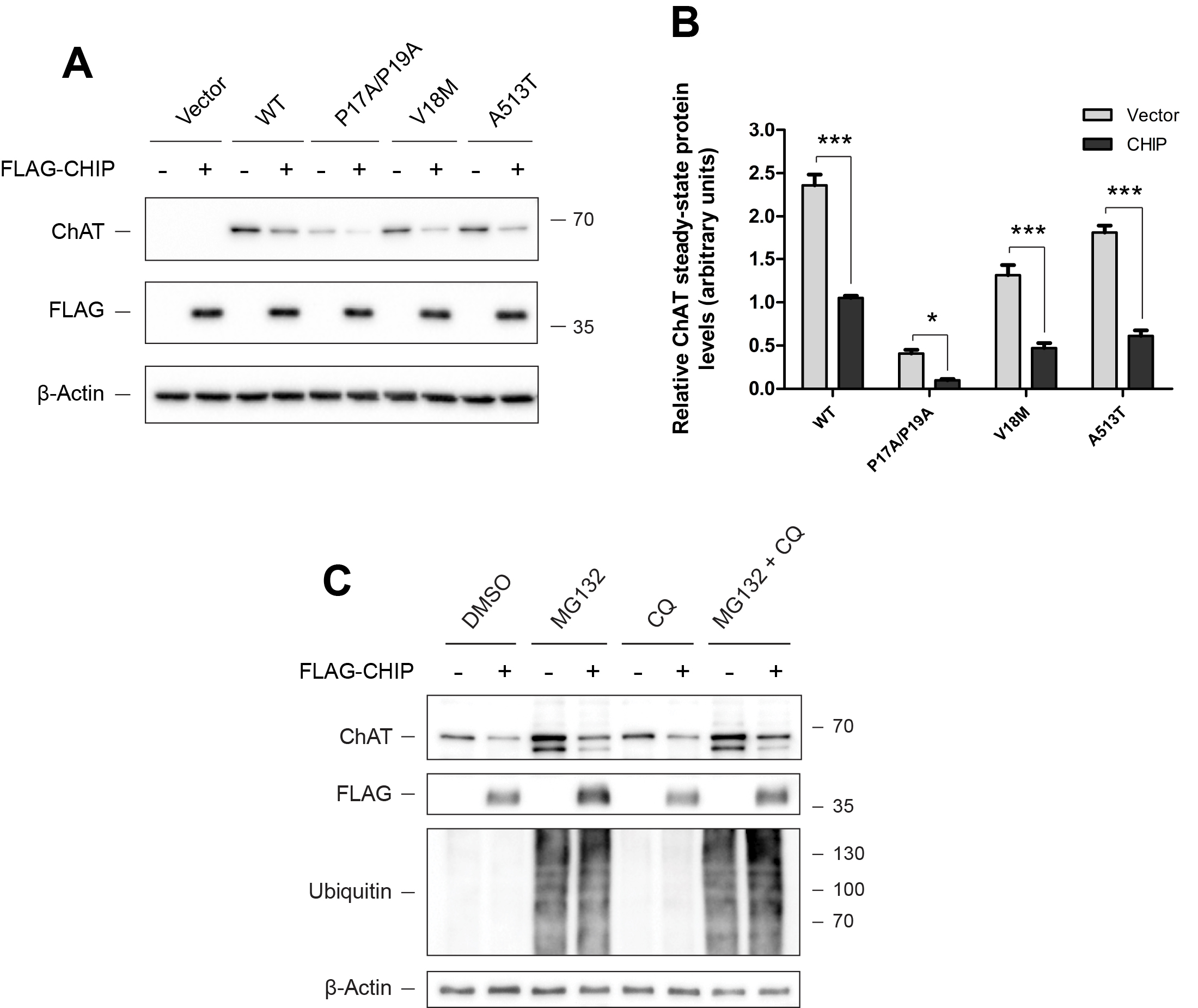

Supplement: Supplementary file 5 [file Image5.JPEG]
