# Supplementary material for: Chaperone-Mediated Regulation of Choline Acetyltransferase Protein Stability and Activity by HSC/HSP70, HSP90, and p97/VCP
Source: Front Mol Neurosci. 2017 Dec 12;10:415. doi: 10.3389/fnmol.2017.00415 (PMC5733026; doi:10.3389/fnmol.2017.00415)
Supplement: Supplementary file 6 [file DataSheet1.docx]

**SUPPLEMENTARY INFORMATION**

**MATERIALS AND METHODS:**

**CHIP overexpression**

To determine the effect of CHIP overexpression on ChAT steady-state protein levels, SN56 cells were transfected to express wild-type or mutant ChAT (P17A/P19A-, V18M, or A513T-ChAT) alone or in combination with FLAG-tagged CHIP. Control cells expressing either wild-type or mutant ChAT alone were co-transfected with empty vector (pcDNA3.1+) to maintain equal amounts of transfected plasmid DNA between samples. Cells were collected and lysed on ice in RIPA buffer (50 mM Tris-HCl; pH 8.0, 150 mM NaCl, 1% Triton X-100, 0.5% sodium deoxycholate, 0.1% SDS) supplemented with mammalian protease inhibitor cocktail (Sigma), phosphatase inhibitor cocktail (10 mM NaF, 1 mM Na_3_VO_4_, 20 mM Na_2_HPO_4_, 3 mM β-glycerolphosphate, 5 mM sodium pyrophosphate), 50 µM MG132 (Enzo Life Sciences), 10 mM N-ethylmaleimide (NEM; Calbiochem), and 800 U/ml DNase I (Invitrogen). Lysates were centrifuged for 10 min at 21,000 ***g*** at 4°C, denatured in 1x Laemmli sample buffer (63 mM Tris-HCl; pH 6.8, 10% glycerol, 2% SDS, 0.005% bromophenol blue, 2.5% 2-mercaptoethanol) at 95°C for 10 min, then analyzed by immunoblotting with anti-ChAT primary antibody.

In some experiments, effects of either proteasome or lysosome inhibition on ChAT steady-state protein levels during heterologous expression of CHIP were examined. SN56 cells expressing either wild-type or mutant ChAT alone or with FLAG-CHIP were treated for 18 h with either 5 µM MG132 or 50 µM chloroquine or a combination of the two. Cells were collected on ice, boiled/lysed in 1% SDS lysis buffer (50 mM Tris-HCl; pH 8.0, 150 mM NaCl, 1% SDS) supplemented with protease/phosphatase inhibitors, 50 µM MG132 and 10 mM NEM, then samples were prepared for anti-ChAT immunoblotting as above. Proteasome inhibition by MG132 treatment was validated by immunoblotting for the accumulation of ubiquitinated cellular proteins.

**SUPPLEMENTARY FIGURE LEGENDS:**

**Supplementary Figure S1: MALDI-TOF-MS data for HSP70 following BioID. (A)** Peptide coverage map of human heat shock 70 kDa protein 1B (HSP70; NCBIprot database, RefSeq: NP_005337.2) following MALDI-TOF-MS from BioID samples (Fig. 1b). A total sequence coverage of 30% was obtained. **(B)** Peptide list of 20 unique peptides with indicated modifications and relevant MALDI-TOF-MS data for HSP70. One peptide (bordered) was identified with a mass-shift correlating with biotinylation of residue Lys^507^ of human HSP70.

**Supplementary Figure S2: MALDI-TOF-MS data for ChAT-BirA* fusion protein following BioID. (A)** Peptide coverage map of human 69-kDa choline acetyltransferase (ChAT; NCBIprot database; GenBank: AAI30618.1) following MALDI-TOF-MS from BioID samples (Fig. 1b). A total sequence coverage of 31% was obtained. **(B)** Peptide list of 23 unique peptides with indicated modifications and relevant MALDI-TOF-MS data for ChAT. One peptide (bordered) was identified with a mass-shift correlating with biotinylation of residue Lys^4^ of human ChAT. **(C)** Peptide coverage map of bacterial BirA (NCBIprot database; GenBank: AEP13897.1) following MALDI-TOF-MS from BioID samples (Fig. 1b). A total sequence coverage of 42% was obtained. **(D)** Peptide lists of 16 unique peptides with indicated modifications and relevant MALDI-TOF-MA data for bacterial BirA.

**Supplementary Figure S3: LC-ESI-MS/MS data for HSP90 following BioID. (A)** Peptide coverage map of human heat shock protein HSP 90-beta isoform c (HSP90; NCBInr_new database, RefSeq: NP_001258901.1) following LC-ESI-MS/MS from BioID samples (Fig. 1b). A total sequence coverage of 16% with 10 unique peptides with indicated modifications was obtained. **(B)** Peptide list with relevant LC-ESI-MS/MS data for human HSP90.

**Supplementary Figure S4: LC-ESI-MS/MS data for HSP70 following BioID.** **(A)** Peptide coverage map of human heat shock 70 kDa protein 1B (HSP70; NCBInr_new database, RefSeq: NP_005337.2) following LC-ESI-MS/MS from BioID samples (Fig. 1b). A total sequence coverage of 34% with 25 unique peptides with indicated modifications was obtained. **(B)** Peptide list with relevant LC-ESI-MS/MS data for human HSP70.

**Supplementary Figure S5: CHIP overexpression promotes proteasomal degradation of ChAT. (A)** Immunoblots from SN56 cells expressing wild-type or mutant ChAT either alone or with FLAG-tagged CHIP co-expression. Control cells were transfected with either empty vector or to express FLAG-CHIP alone. **(B)** Steady-state proteins levels of wild-type (***p≤0.001), P17A/P19A- (*p≤0.05), V18M- (***p≤0.001), and A513T-ChAT (***p≤0.001) are reduced following overexpression of FLAG-CHIP as compared to cells co-transfected with equal amounts (µg) of empty vector (two-way ANOVA with Bonferroni’s *post-hoc* test, mean ± SEM, *n*=4). **(C)** Proteasome inhibition partially attenuates loss of ChAT following overexpression of FLAG-CHIP. Immunoblots from SN56 cells co-expressing wild-type ChAT with FLAG-tagged CHIP that were treated with either 5 µM MG132 or 50 µM chloroquine (CQ) alone or in combination for 18 h. Control cells were treated with DMSO-vehicle and/or were transfected with either empty vector or to express wild-type ChAT alone (*n*=6).
